# Organotypic whole hemisphere brain slice models to study the effects of donor age and oxygen-glucose-deprivation on the extracellular properties of cortical and striatal tissue

**DOI:** 10.1186/s13036-022-00293-w

**Published:** 2022-06-13

**Authors:** Michael McKenna, Jeremy R. Filteau, Brendan Butler, Kenneth Sluis, Michael Chungyoun, Nels Schimek, Elizabeth Nance

**Affiliations:** 1 Department of Chemical Engineering, University of Washington, 105 Benson Hall, Box 351750, Seattle, WA 98195-1750 USA; 2 Department of Chemistry, University of Washington, Seattle, WA USA; 3 e-Science Institute, University of Washington, Seattle, WA USA; 4 Department of Bioengineering, University of Washington, Seattle, WA USA

**Keywords:** Brain slices, Organotypic, Extracellular, Brain microenvironment, Nanoparticle diffusion, Particle tracking

## Abstract

**Background:**

The brain extracellular environment is involved in many critical processes associated with neurodevelopment, neural function, and repair following injury. Organization of the extracellular matrix and properties of the extracellular space vary throughout development and across different brain regions, motivating the need for platforms that provide access to multiple brain regions at different stages of development. We demonstrate the utility of organotypic whole hemisphere brain slices as a platform to probe regional and developmental changes in the brain extracellular environment. We also leverage whole hemisphere brain slices to characterize the impact of cerebral ischemia on different regions of brain tissue.

**Results:**

Whole hemisphere brain slices taken from postnatal (P) day 10 and P17 rats retained viable, metabolically active cells through 14 days in vitro (DIV). Oxygen-glucose-deprivation (OGD), used to model a cerebral ischemic event in vivo, resulted in reduced slice metabolic activity and elevated cell death, regardless of slice age. Slices from P10 and P17 brains showed an oligodendrocyte and microglia-driven proliferative response after OGD exposure, higher than the proliferative response seen in DIV-matched normal control slices. Multiple particle tracking in oxygen-glucose-deprived brain slices revealed that oxygen-glucose-deprivation impacts the extracellular environment of brain tissue differently depending on brain age and brain region. In most instances, the extracellular space was most difficult to navigate immediately following insult, then gradually provided less hindrance to extracellular nanoparticle diffusion as time progressed. However, changes in diffusion were not universal across all brain regions and ages.

**Conclusions:**

We demonstrate whole hemisphere brain slices from P10 and P17 rats can be cultured up to two weeks in vitro. These brain slices provide a viable platform for studying both normal physiological processes and injury associated mechanisms with control over brain age and region. Ex vivo OGD impacted cortical and striatal brain tissue differently, aligning with preexisting data generated in in vivo models. These data motivate the need to account for both brain region and age when investigating mechanisms of injury and designing potential therapies for cerebral ischemia.

**Supplementary Information:**

The online version contains supplementary material available at 10.1186/s13036-022-00293-w.

## Background

The brain extracellular microenvironment is involved in many critical processes associated with neurodevelopment and neuronal health throughout life and in aging [1–3]. It consists of an extracellular space (ECS) that contains a dynamic milieu of ions and signaling molecules and a proteoglycan-based scaffold known as the extracellular matrix (ECM) [4, 5]. The anionic composition of brain ECM allows it to sequester and present ions and neurotransmitters to nearby cells in a controlled manner [6–9]. Brain ECM also helps maintain proper cellular spacing by imbibing water and providing cells with tethering points. An intriguing property of both brain ECS and brain ECM is that they are structurally dynamic. The ECS can undergo volumetric changes during hyperexcitatory discharge events and during sleep, where a circadian volume-change rhythm is used to clear metabolites as part of the glymphatic system [10–12]. The ECM can degrade, rearrange, or condense in response to shifts in neuronal firing patterns during long-term potentiation and depression [13, 14]. Despite growing awareness of the critical roles of the brain extracellular environment in many aspects of neural function, the variability of both ECS and ECM across different brain regions is not well-understood. Some work has characterized differential patterns of ECM protein expression and quantified the density of ECM structures across different brain regions [15, 16], but these studies leveraged fluorescence-based labeling of specific ECM components, which provide little insight into ECS geometry or transport properties. Diffusion-based techniques have been performed in different brain regions to assess regional differences in ECS transport properties [5, 17, 18], but work to-date has used volume-averaging methods that fail to capture the true heterogeneity of brain ECS at the microscale. Because of this, there exists no regional ECS structure-function relationships attributed to specific brain areas [12].

**Fig. 1:**
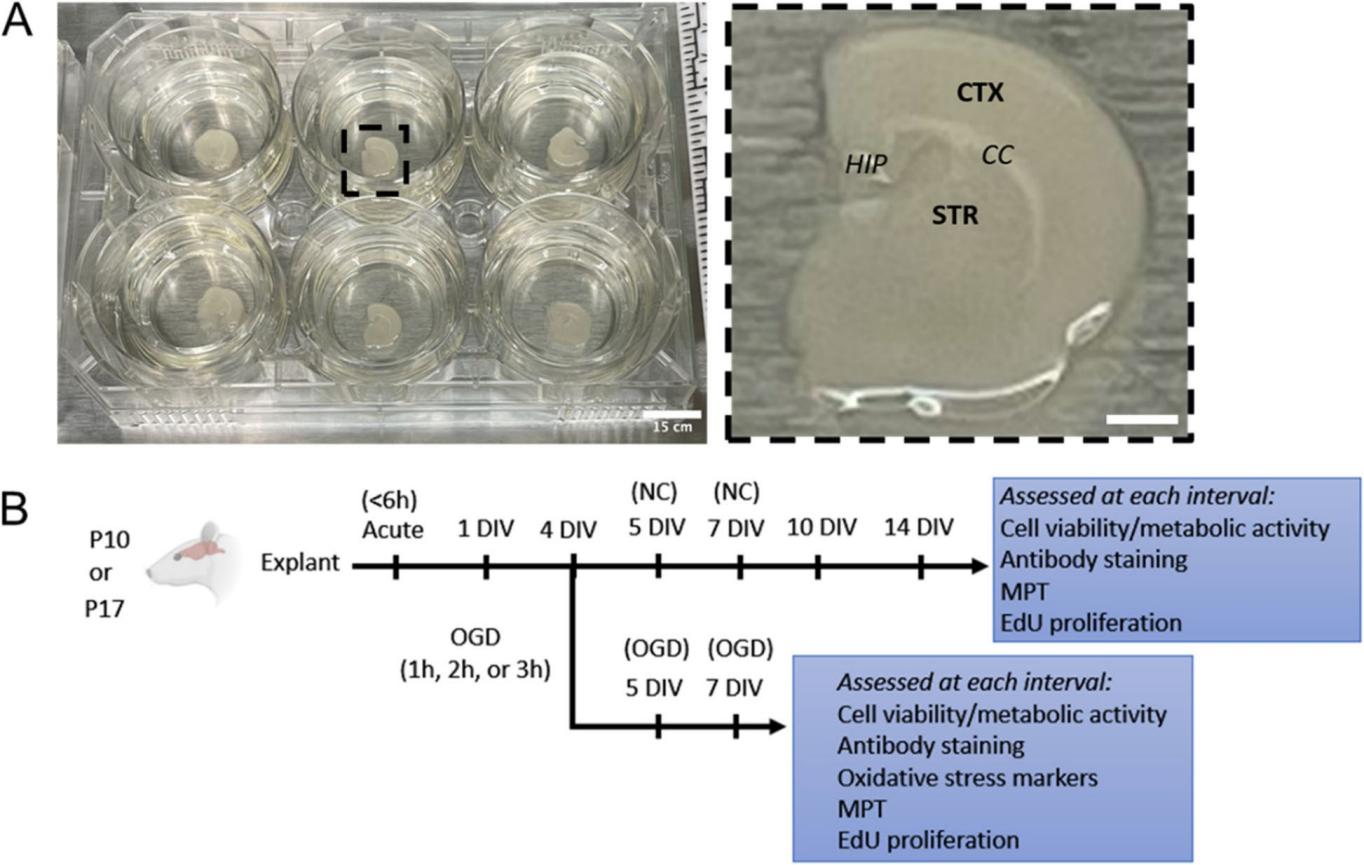
Schematic of OWH slice culture methodology and experimental workflow. **A** Left: Coronal slices containing cortex (CTX) and striatum (STR) are plated onto PTFE membrane inserts. Right: a single OWH brain slice after explantation. Although not imaged in this study, hippocampal (HIP) and corpus callosum (CC) regions are also labeled for reference. Scale bars = 15 cm and 5 cm. **B** Experimental workflow. Slices from postnatal (P) day 10 or P17 aged rat donors are cultured up to 14 days, performing OGD on 4 DIV with endpoints at 5 days in vitro (DIV) (24 h post-OGD), 7 DIV (72 h post-OGD), and 10 DIV

We have recently demonstrated that multiple particle tracking (MPT) is capable of detecting dynamic changes in brain ECM microstructure and quantifying both ECS transport properties and geometry with submicron resolution [19]. Therefore, MPT can be a viable option to study regional differences in the brain extracellular microenvironment. However, since MPT relies on fluorescence microscopy, which limits the depth at which imaging can be performed [20, 21], assessing regional variability requires a platform that provides direct access to multiple brain regions. Organotypic brain slices represent a potential solution, as they preserve functional relationships between neighboring cells and maintain 3D cytoarchitecture [14, 22, 23], a critical feature when trying to probe ECS in an in vivo like state. Organotypic brain slices also provide direct access to multiple regions within brain tissue, including deep brain regions like the striatum. Most work to-date has been performed in brain slices that isolate and culture single brain regions, like the hippocampus or cortex [24–28]. There are fewer instances of whole hemisphere brain slices being used to study brain function or disease processes, and they are typically only acquired from neonatal (<P14) donors [22, 29–32]. Given the changes that occur in brain ECM throughout neurodevelopment, generating slices from different aged donors would be useful [33]. Additionally, to enable longer-term assessment of ECS and ECM changes in response to injury, brain slices must also stay viable for extended periods of time post-slice preparation. We establish an organotypic whole hemisphere brain slice (OWH) platform from two different aged donors that retains slice viability for at least two weeks in vitro to provide greater insight into the temporal effect of culturing time and injury exposure on ECM and ECS dynamics (Fig. 1). For our injury model, we expose OWH slices to oxygen-glucose deprivation (OGD), which models an ischemic injury in vivo [34]. We apply MPT to analyze changes in diffusion to further understand microenvironmental responses to donor age, culturing time, brain region, and OGD exposure.

## Results

### OWH brain slices prepared from both P10 and P17 donors retain viable, metabolically-active cells for 14 days in vitro

**Fig. 2:**
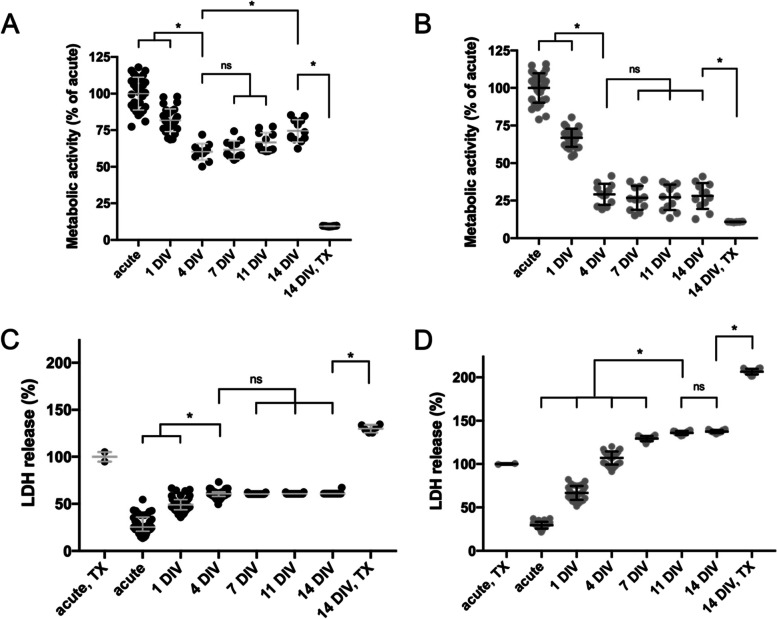
Metabolic activity and LDH release profiles for P10 and P17 brain slices for 14 DIV following explantation. Metabolic activity values are normalized to the metabolic activity at the acute timepoint. LDH release (%) values are normalized to the LDH release of acute slices immediately treated with TX-100. **A-B** Metabolic activity of (**A**) P10 and (**B**) P17 slices. Error bars represent the mean ± SD. **C-D** % LDH release profile of (**C**) P10 and (**D**) P17 slices. Error bars represent median ± IQR (**C**) and mean ± SD (**D**). *n* = 6–42 OWH slices. * denotes significant differences (*p* < 0.05). For metabolic activity data (**A**&**B**) and LDH release data in P10 brain slices (**C**), comparisons were made between the 4DIV timepoint and all other time points. For LDH release data in P17 brain slices (**D**), comparisons were made between the 11DIV timepoint and all other time points. In all instances (**A-D**), comparisons were also made between the 14DIV timepoint and the 14DIV, TX-100 treated group

To determine if we could effectively culture OWH brain slices taken from both postnatal (P) day 10 and P17 rats, slice metabolic activity and viability were monitored over 14 days in vitro (DIV); evaluations were made at an acute timepoint, then subsequently on 1DIV, 4DIV, 7DIV, 11DIV, and 14DIV. The metabolic activity of both P10 and P17 slices decreased over the first 4DIV, with P17 slices decreasing more significantly (Fig. 2A-B). The mean (± SD) metabolic activity of P17 slices on 4DIV was 29.2% (± 7.0%), compared to 60.2% (± 5.5%) for P10 slices. After 4DIV, the metabolic activity of P10 slices began to increase and continually increased through 14DIV (Fig. 2A). No significant differences in metabolic activity were observed in P17 slices after 4DIV (Fig. 2B), indicating a stable level of whole slice metabolic activity had been reached. For both age groups, the positive control Triton X (TX)-100 treatment at 14DIV resulted in significantly lower metabolic activity than was observed at any other timepoint (Fig. 2A-B).

At the same timepoints, cell death was quantified by measuring the concentration of lactate dehydrogenase (LDH) released into the culture media. The amount of LDH released by slices immediately treated with TX-100 was used as the 100% LDH release control, and cumulative LDH release (%) starting from the acute timepoint was reported for all other timepoints. Similar to the metabolic activity decrease over the first 4DIV in P10 slices, LDH release increased. Median (IQR) % LDH release increased significantly from 25.3% (20.9–35.9%) at the acute timepoint to 49.0% (43.6–65.5%) and 60.8% (58.0–62.9%) at 1DIV and 4DIV, respectively (Fig. 2C). Cumulative LDH release remained constant after 4DIV. At all timepoints, P17 slices had greater % LDH release than P10 slices (Fig. 2D).

Additionally, in P17 slices, the progressive increase in cumulative % LDH release lasted further into the 14-day experimental window and even exceeded the 100% LDH release control (Fig. 2D). % LDH release significantly increased from acute to 11DIV. Cumulative LDH release did not increase significantly from 11DIV to 14DIV, where it settled at 138% (± 2%). Slices were treated with TX-100 at 14DIV to provide a positive cell death control at the study endpoint. TX-100 treatment at 14DIV resulted in a significant increase in % LDH release in both age groups (Fig. 2C-D).

### Propidium iodide (PI) staining provides regional assessment of viability in P10 and P17 brain slices

**Fig. 3:**
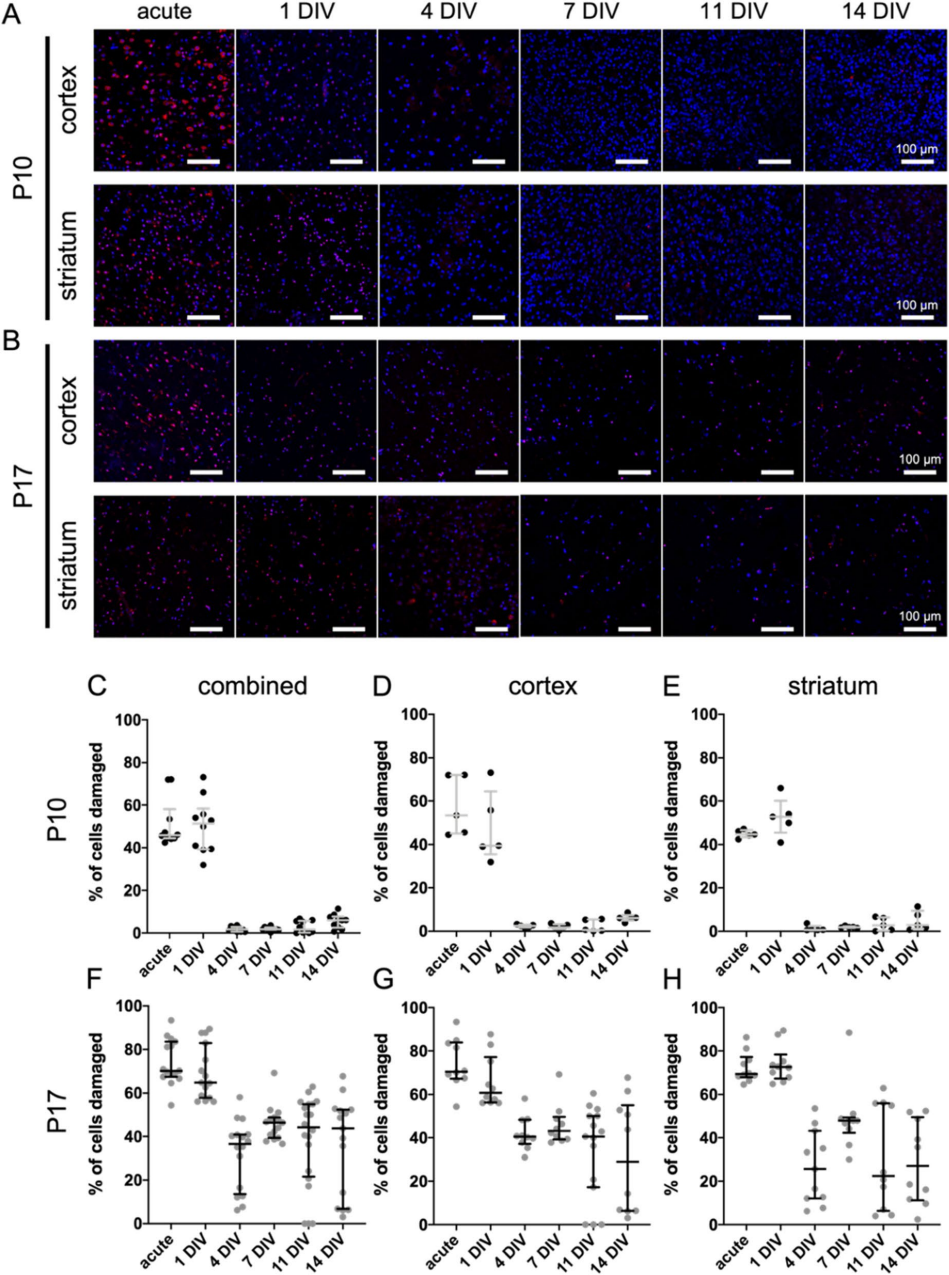
Propidium iodide imaging in normal control P10 and P17 slices through 14DIV. Representative 40x images of either (**A**) P10 or (**B**) P17 brain slices taken at acute, 1DIV, 4DIV, 7DIV, 11DIV, and 14DIV timepoints. Images are split into two rows for each age. Top row: cortex; bottom row: striatum. Error bars: 100 μm. **C-H** Quantification of propidium iodide imaging in normal control P10 and P17 slices through 14DIV. Profiles for P10 slices are given for (**C**) all data combined, (**D**) the cortex, and (**E**) the striatum. P17 profiles are given for (**F**) all data combined, (**G**) the cortex, and (**H**) the striatum. Error bars represent the median ± IQR

We leverage PI, a fluorescence-based imaging approach for quantifying cell damage [35, 36], to characterize how both the cortical and striatal regions of P10 and P17 OWH slices respond to the transition ex vivo. The cortex and striatum play critical roles in normal brain function [37–40] and are susceptible to hypoxic-ischemic events like cerebral ischemia [41–43], which we model using OGD. Representative images taken from the cortex and striatum – specifically the caudate putamen – of P10 and P17 brain slices reveal a similar trend as the alamarBlue and LDH assays: the P10 slices recover quicker from the initial damage induced by slice preparation; this trend was consistent regardless of brain region (Fig. 3A-B). Qualitatively, the % of cells damaged in P10 slices, both in the cortex and striatum, was greatest at the acute and 1DIV timepoints, as evidenced by high numbers of DAPI-positive (blue) cell nuclei that were also PI-positive (red) (Fig. 3A). PI signal was diminished by 4DIV and remained low at all remaining timepoints out to 14 DIV. These qualitative trends were confirmed upon quantification (Fig. 3C-H). In the cortex of P10 slices, the median (IQR) % of cells damaged was 39.5% (35.5–64.5%) at 1DIV, before dropping significantly (Fig. 3D). Likewise, in the striatum, the median % of cells damaged was 52.9% (45.5–60.1%) at 1DIV, then decreased significantly (Fig. 3E).

In P17 slices, the proportion of DAPI-positive nuclei that were also PI-positive remained more consistent over the 14-day culture period (Fig. 3B). The acute and 1DIV timepoints in P17 slices contained the greatest overlap between DAPI and PI signal qualitatively, regardless of brain region. However, unlike in P10 slices, a notable % of cells in P17 slices remained damaged at later DIV timepoints in both regions (4DIV, 7DIV, 11DIV, and 14DIV) (Fig. 3B). Quantifying the % of cells damaged confirmed these observations. In both the cortex and striatum of P17 slices, the % of cells damaged was greatest at the acute and 1DIV timepoints (Fig. 3F-H). In the cortex, median (IQR) % of cells damaged was 70.5% (67.3–83.9%) and 60.8% (56.3–77.1%) at acute and 1DIV, respectively (Fig. 3G). The % of cells damaged in the striatum at acute and 1DIV was 69.4% (67.9–77.2%) and 72.6% (67.3–78.3%), respectively (Fig. 3H).

In the cortex, the median % of cells damaged decreased and was no greater than 43.2% at 4DIV, 7DIV, 11DIV, and 14DIV. The median % of cells damaged in the striatum was no greater than 47.9% at any of the timepoints after 1DIV. Unlike P10 slices, however, the median % of cells damaged never fell below 22.4% in either the cortex or striatum of P17 slices (Fig. 3F-H). Additionally, the outcomes in P17 slices were more variable. In P10 slices, at all timepoints after 1DIV, the distribution of the % of cells damaged was narrower than it was in P17 slices, and the 75% percentile never exceed 9.48% (Fig. 3C-E). In P17 slices, the distribution of data, especially at 4DIV, 7DIV, 11DIV, and 14DIV, was wider. In some images, the % of cells damaged was less than 5%. In others, the % of cells damaged remained above 40% (Fig. 3F-H).

Lastly, in P10 slices, notable differences in the total number of cells present existed at each timepoint (Fig. 3A). The lowest cell density occurred qualitatively at 4DIV in both the cortex and striatum and in both regions appeared to increase at later timepoints. Qualitatively, there was a higher cell density at 7DIV, 11DIV, and 14DIV than there was at 4DIV (Fig. 3A). This trend was not observed in P17 slices. The cell density in P17 slices was greatest at the acute timepoint, then decreased to a level at 4DIV where it remained relatively constant for the remainder of the 14-day culture window (Fig. 3B). We measured cellular proliferation via 5-ethynyl-2′-deoxyuridine (EdU) staining [44–46]. EdU and BrdU have been used extensively to measure proliferation, but we used EdU to avoid the harsh antigen retrieval steps necessary for detecting the antibody to BrdU after fixation [46]. Less than 5% Edu + cells were detected in P10 and P17 slices at 10 DIV (Fig. S1A), and a detectable amount of proliferation was only detected in the striatum at 10DIV of P10 slices (Fig. S1B).

### MPT provides a regional assessment of brain extracellular transport properties in normal control (NC) P10 and P17 OWH brain slices

**Fig. 4:**
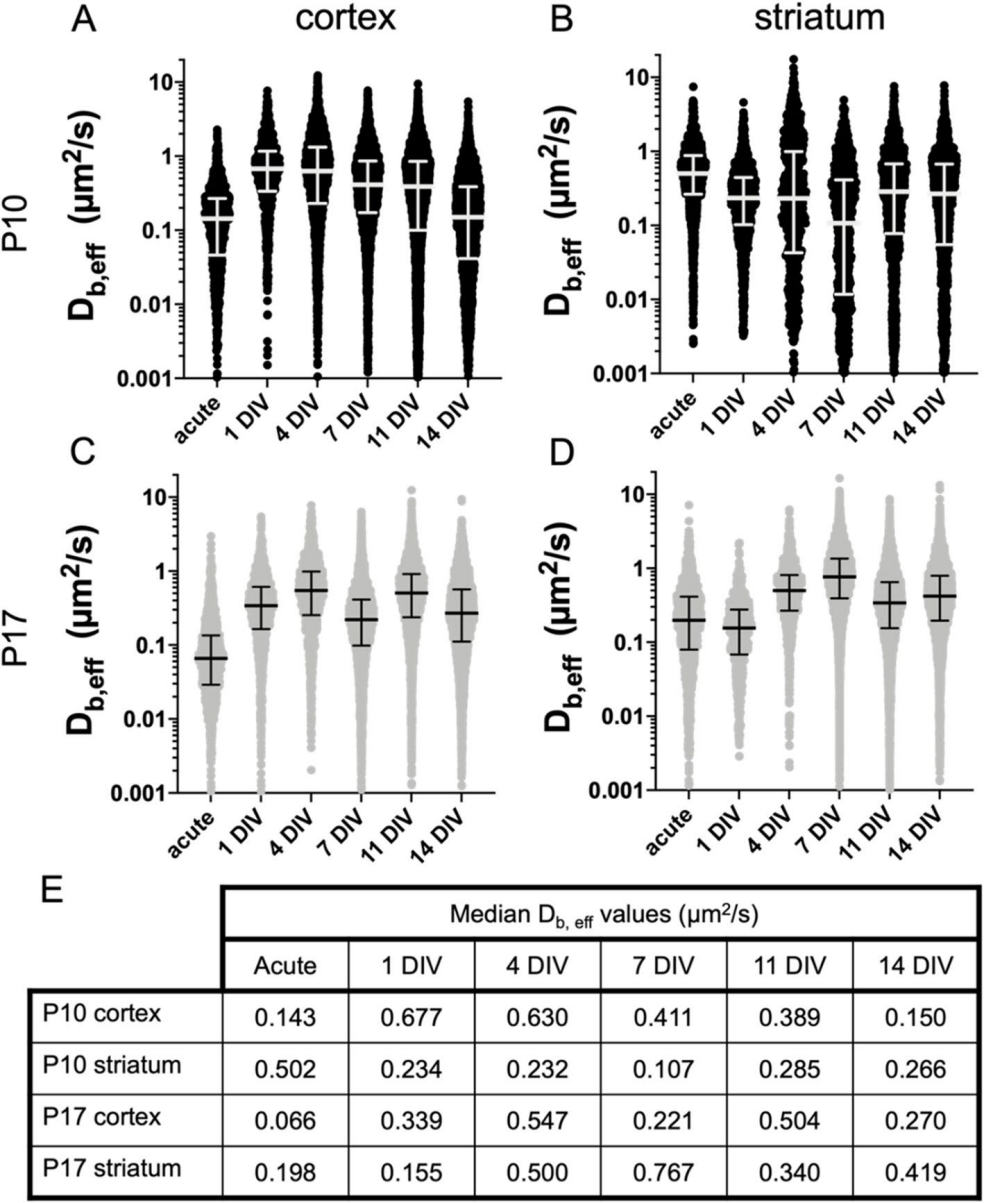
MPT in P10 and P17 NC brain slices through 14DIV. D_b,eff_ distributions at acute, 1DIV, 4DIV, 7DIV, 11DIV, and 14DIV. Data is provided for the (**A**) cortex of P10 slices, (**B**) striatum of P10 slices, (**C**) cortex of P17 slices, and (**D**) striatum of P17 slices. For D_b,eff_ distributions, error bars represent the median value and IQR. **E** Median D_b, eff_ values are provided for each age and brain region at each timepoint

To characterize how the brain extracellular environment changes following explantation, we performed MPT with 40 nm PS-PEG nanoparticles at acute, 1DIV, 4DIV, 7DIV, 11DIV, and 14DIV in both the cortex and striatum of P10 and P17 OWH brain slices. Nanoparticle physicochemical properties are provided in Table S1. Nanoparticle trajectories were quantified using a custom Python package [47] that leverages TrackMate, an open source Fiji plugin for the tracking of single particles [48]. From the raw trajectories, trajectory mean square displacements (MSD) were calculated at various lag times and the Einstein-Smoluchowski relation was used to obtain effective diffusion coefficients in the brain (*D*_b,eff_) for each nanoparticle trajectory in a given population [49, 50]. Distributions of *D*_b,eff_ values at each timepoint, split by brain region and age, are displayed in Fig. 4.

In the cortex of both P10 and P17 slices, the median *D*_b,eff_ value was lowest at the acute timepoint then increased to a max value within the next two timepoints; median *D*_b,eff_ reached a max value of 0.677 μm^2^/s at 1DIV in P10 slices and a max value of 0.547 μm^2^/s at 4DIV in P17 slices (Fig. 4A,C). In the cortex of P10 slices, the particle population progressively slowed after hitting its max value at 1DIV. In the cortex of P17 slices, the same progressive decline was not observed after reaching its max: despite dipping at 7DIV, the median *D*_b,eff_ value at 11DIV (0.504 μm^2^/s) was not significantly different than the median *D*_b,eff_ value observed at 4DIV.

In the striatum, less discernable trends in *D*_b,eff_ distributions were present, both in P10 and P17 OWH slices (Fig. 4B,D). The behavior of nanoparticle populations in the striatum of P17 slices did follow a similar trend to what was observed in the cortex of both P10 and P17 slices, but the trend was delayed. The slowest moving particle population occurred at 1DIV in the striatum of P17 slices (median *D*_b,eff_ value of 0.155 μm^2^/s), as opposed to the acute timepoint in the cortex of both P10 and P17 slices. The median *D*_b,eff_ value then hit its max of 0.767 μm^2^/s at 7DIV; it occurred at 1DIV and 4DIV in the cortex of P10 and P17 slices, respectively (Fig. 4D). Finally, the behavior of nanoparticle populations in the striatum of P10 slices was unlike any other region in either age group. The particle populations slowed progressively from acute to 7DIV, before increasing to a level at 11DIV (median *D*_b,eff_ of 0.285 μm^2^/s) that it remained close to for the final, 14DIV timepoint (median *D*_b,eff_ of 0.266 μm^2^/s at 14 DIV–not significantly different than the 11DIV timepoint) (Fig. 4B).

In addition to *D*_b,eff_ distributions, the anomalous diffusion exponent, α, was generated by fitting MSD versus time curves to the anomalous diffusion equation. Trajectories were categorized as either immobile (α < 0.1), subdiffusive (0.1 ≤ α < 0.9), normally diffusive (0.9 ≤ α ≤1.1), or superdiffusive (1.1 < α). The % of particles at a given time point exhibiting each of the four motion types was plotted over 14 days (Fig. S2). The trends in diffusion modes observed in the cortex of P10 slices aligned with *D*_b,eff_ distribution trends. Where *D*_b,eff_ distributions were shifted to smaller *D*_b,eff_ values at acute and 14DIV, the % of particles exhibiting either immobile or subdiffusive behavior were at their greatest (Fig. S1A). Similarly, where *D*_b,eff_ distributions were shifted to larger *D*_b,eff_ values at 1DIV, 4DIV, and 7DIV, the % of particles exhibiting either normal diffusion or superdiffusive behavior increased. In the striatum of P10 slices, the % of particles exhibiting subdiffusive behavior was stable throughout, fluctuating between 41.2 and 43.9% (Fig. S1B). The % of particles diffusing normally or exhibiting superdiffusive behavior decreased over time (Fig. S1B). In P17 slices, the trends in diffusion modes were similar in both regions (Fig. S1C-D). In both the striatum and cortex, the highest % of particles that were immobile occurred at either the acute or 1DIV timepoint. Again, in both the cortex and striatum, the % of particles exhibiting either normal diffusion or superdiffusive behavior increased from acute to 1DIV to 4DIV, before stabilizing for the remainder of the 14-day window (Fig. S1C-D).

### The effect of OGD on whole slice viability and metabolic activity is not age-dependent, but the response to OGD was more regionally variable in P17 brain slices than P10

**Fig. 5:**
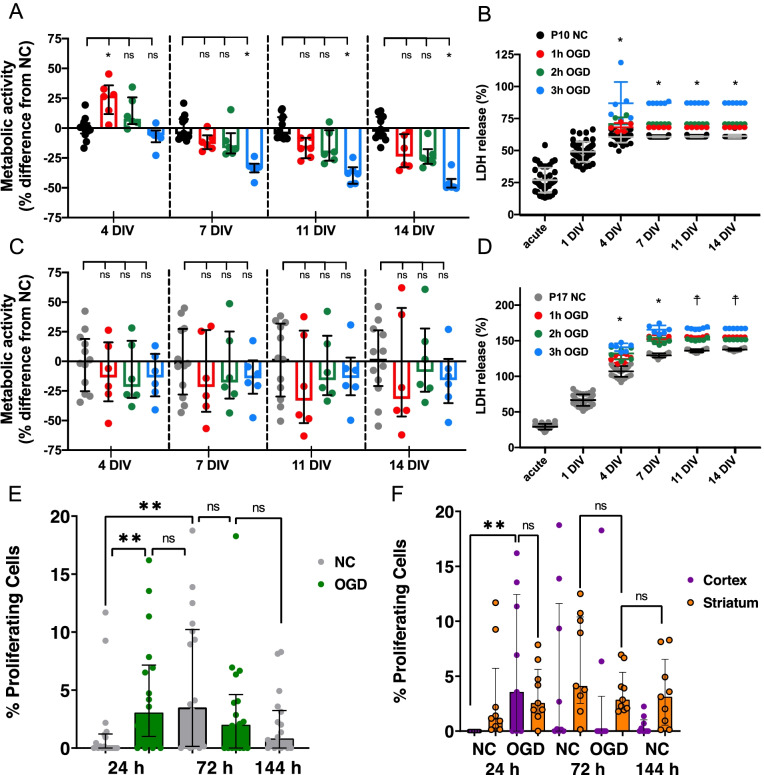
Metabolic activity, LDH release, and cell proliferation profiles following OGD. **A** Metabolic activity of P10 slices after 1 h, 2 h, or 3 h OGD. Error bars represent median and IQR. **B** LDH release (%) profiles for P10 slices after 1 h, 2 h, or 3 h OGD. Error bars represent mean ± SD. **C** Metabolic activity profiles for P17 slices after 1 h, 2 h, or 3 h OGD. Error bars represent median and IQR. * denotes significant differences (*p* < 0.05) between all OGD groups (1 h, 2 h, and 3 h) and the NC. **D** LDH release (%) profiles for P17 slices after 1 h, 2 h, or 3 h OGD. Error bars represent mean ± SD. * denotes significant differences (*p* < 0.05) between all OGD groups (1 h, 2 h, and 3 h) and the NC. ☨ denotes significant differences (*p* < 0.05) between the 1 h and 3 h OGD groups and the NC. For (**A**) and (**C**), *n* = 6–12 slices per condition. For (**B**) and (**D**), *n* = 6 slices for all OGD conditions, and *n* = 12–42 slices for NC conditions. Each data point represents a single slice. **E** Percentage of proliferating cells in P10 slices throughout culture and after 2 h OGD. 24 h time points refer to time after OGD, equivalent to 5DIV; 72 h time points refer to 72 h after OGD, equivalent to 7DIV. Data from a NC slice at 144 h (10DIV) is provided for comparison. **F** Percentage of proliferating cells in the cortex and striatum following 2 h OGD and compared to NC. Each data point represents a single image from 3 total slices per group

We next sought to investigate the effect of slice donor age on susceptibility to a hypoxic*-*ischemic event. We varied the duration of OGD at 4DIV on both P10 and P17 OWH brain slices; the durations of OGD tested at 4DIV were 1 h, 2 h, and 3 h OGD. To quantify the damage induced by OGD, we ran alamarBlue and LDH assays immediately after injury, then subsequently on the same days we previously assessed normal control (NC) slices: at 7DIV, 11DIV, and 14DIV, corresponding to 3, 7, and 11 days post-OGD (Fig. 5). We also assessed cellular response to OGD via imaging of hypoxia inducible factor 1 subunit alpha (HIF1α) and cellular proliferation via EdU staining [44–46].

Interestingly, in P10 slices, the metabolic activity at the acute timepoint was significantly greater than the NC following 1 h OGD (Fig. 5A). 2 h OGD and 3 h OGD altered metabolic activity compared to NC levels, but not significantly. At 3, 7, and 11 days post-OGD, P10 whole-slice metabolic activity was reduced in an OGD-duration-dependent manner (Fig. 5A), with only 3 h OGD resulting in a significant reduction in metabolic activity compared to the NC. The median metabolic activity in all P17 OGD groups was always less than in NC slices (Fig. 5C). None of the differences were significant at any timepoints, and no OGD-duration-dependencies existed. The metabolic activity of P17 NC slices was more variable than in P10 NC slices, providing a potential explanation as to why no significant differences existed in P17 OGD groups (Fig. 5A,C).

LDH revealed an increase in % LDH release in both P10 and P17 OWH brain slices following OGD, regardless of OGD duration (Fig. 5B,D). The increase in LDH release was OGD-duration-dependent in P10 slices; median % LDH release at 14DIV for 1 h, 2 h, and 3 h OGD groups was 68.6, 71.3, and 87.6%, respectively, compared to 60.9% for the NC group (Fig. 5B). While not dependent on OGD-duration, cumulative % LDH release in P17 slices was significantly greater in all OGD groups compared to NC slices at both the acute (4DIV) and 3-days post-OGD timepoints (7DIV) (Fig. 5D). The differences in % LDH release persisted to 14DIV (Fig. 5D).

We assessed whether OGD alters cellular proliferation in P10 slices only. Following 2 h OGD, there was a significant increase in the percentage of EdU+ cells 24 h after OGD compared to NC (Fig. 5E). However, by 72 h post-OGD, no significant difference in number of EdU+ cells in OGD and NC was detected. On a per region basis, both cortex and striatum had an increase in percentage of EdU+ cells by 24 h after OGD (Fig. 5F). By 72 h after OGD, the percentage of EdU+ cells in the cortex were back to levels observed in the 24 h NC. However, the striatum responded differently, as the % proliferating cells in the 72 h OGD group is higher than the 24 h NC value, but less than the 72 h NC value, though not significantly higher.

**Fig. 6:**
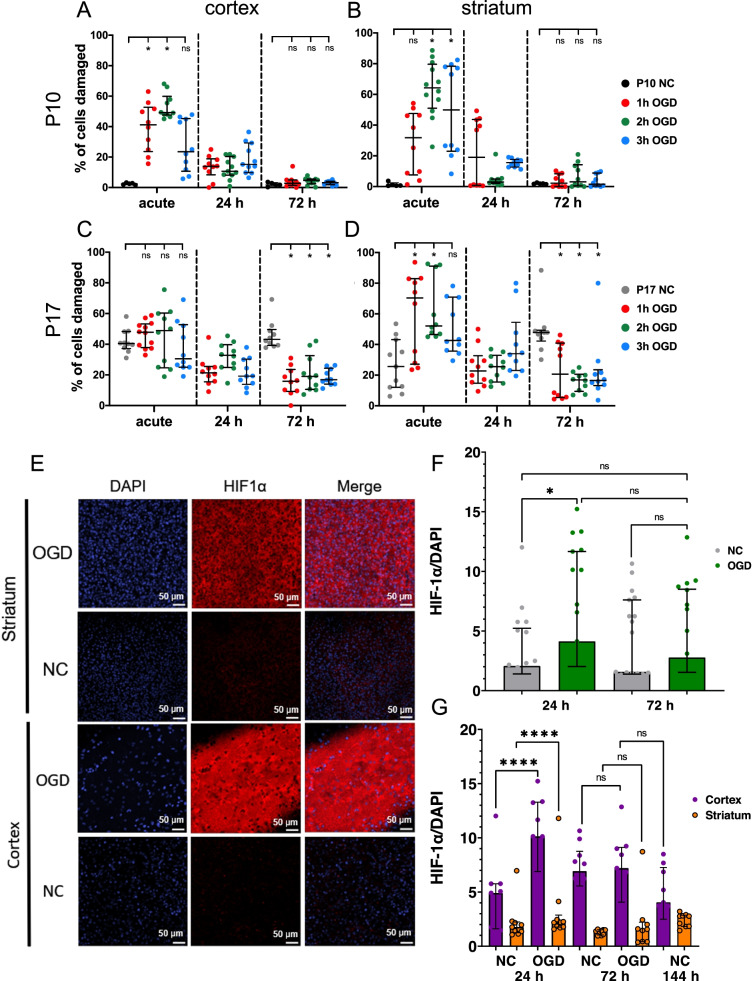
Profiles of % of cells damaged following 1 h, 2 h, or 3 h OGD quantified via PI staining, live-dead cell counting, and HIF1α intensity. Data from each age are contained in their own row. Each dot represents a single ROI, where *n* = 10–12 ROIs with 5–6 ROIs per slice. P10 (**A**) cortex and (**B**) striatum data are presented in the top row. P17 (**C**) cortex and (**D**) striatum data are given in the bottom row. Error bars represent the median ± IQR. **E** Representative images of HIF1α staining 24 h after 2 h OGD at 4DIV from P10 slices (scale bar = 50 μm). **F** Mean intensity of HIF1α staining 24 h and 72 h after 2 h OGD. Each point represents a single image. **G** Mean intensity of HIF1α staining 24 h and 72 h after 2 h OGD in the cortex and striatum. Error bars represent the median ± IQR for (**E**, **F**, **G**)

P10 and P17 slices were assessed regionally at an acute timepoint (4DIV, the day OGD was performed), then at 24 h and 72 h post-OGD for 1 h, 2 h, and 3 h OGD exposure (Fig. 6). In P10 slices, both regions were impacted similarly: the % of cells damaged was elevated compared to the NC at the acute timepoint but returned to NC levels by 72 h post-OGD. In the cortex, both 1 h and 2 h OGD significantly increased the % of cells damaged at the acute timepoint (Fig. 6A). At the acute timepoint in the striatum, 2 h and 3 h OGD significantly increased the % of cells damaged (Fig. 6B). In both regions, none of the OGD groups were significantly different than the NC by 72 h post-OGD. Interestingly, 2 h OGD resulted in the greatest % of cells damaged at the acute timepoint in both regions, and no OGD-duration-dependencies existed. In P17 slices, the striatum was more impacted by OGD than the cortex. In the cortex, none of the OGD durations increased the % of cells damaged at the acute timepoint significantly (Fig. 6C). In the striatum, however, both 1 h and 2 h OGD lead to a significant increase in % cells damaged compared to the NC (Fig. 6D). In both the cortex and striatum of P17 slices at 72 h post-OGD, all OGD groups had a significantly lower % of damaged cells than the NC group (Fig. 6C-D). Similar to P10 slices, no OGD-duration-dependencies existed in either region.

Given that P10 slices retained a higher viability than P17 slices overall throughout the culturing window, we further investigated the effect of OGD on P10 slices by performing immunohistochemistry with HIF1α, a marker for hypoxia, on P10 slices subjected to 2 h of OGD and compared to NC. Endpoints for staining were 24 h and 72 h after OGD and representative images are shown in Fig. 6E. There was a significant increase in HIF1α signal in OGD slices 24 h after 2 h OGD compared to NC; however, this difference was not sustained out to 72 h after 2 h OGD (Fig. 6F). When analyzing HIF1α signal by brain region, there was a significant increase in HIF1α in the cortex after 2 h OGD, but not in the striatum (Fig. 6G).

**Fig. 7:**
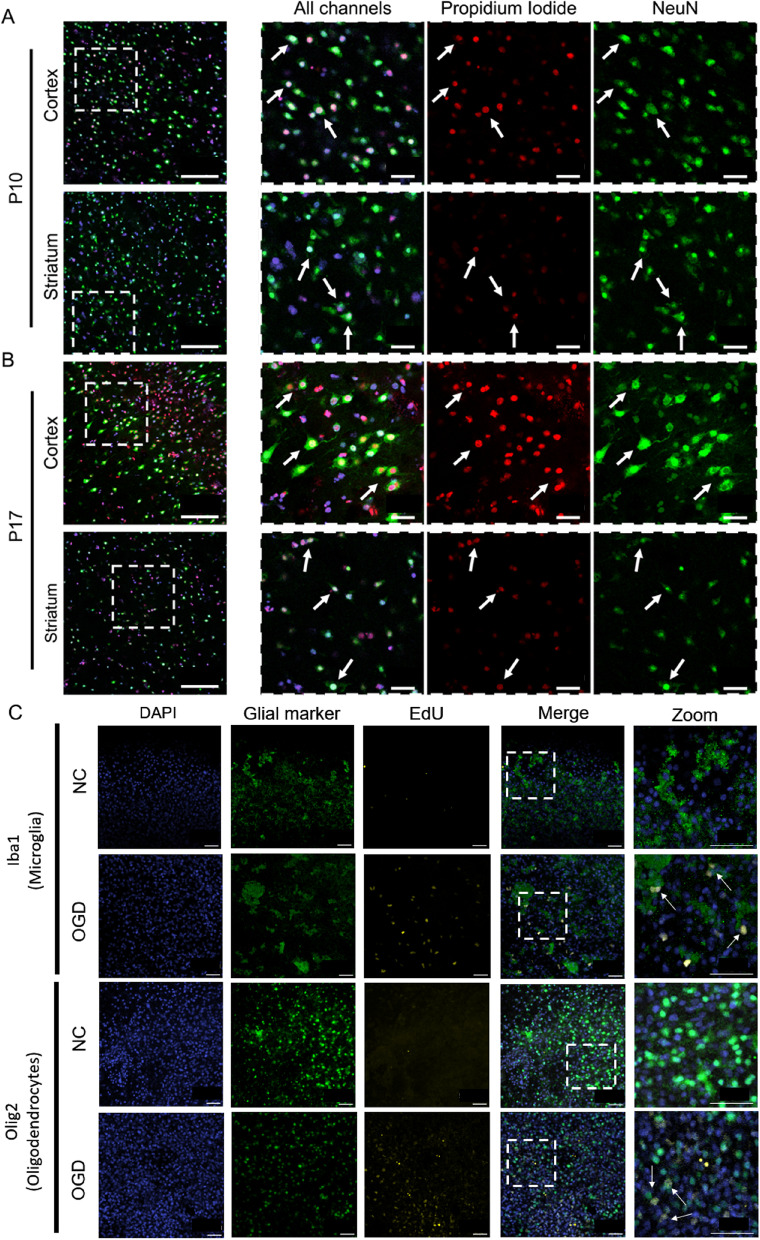
Cellular response immediately following 2 h OGD. Representative images are shown for (**A**) P10 and (**B**) P17 slices for PI (red) and NeuN (green) co-staining. The left column has a 40x magnification image; higher magnification images from the white dashed line box display a merged image, an image of PI alone, and an image of NeuN alone. Scale bars for the 40x magnification images: 100 μm. Scale bars for the zoomed in images: 25 μm. **C** Representative images of Iba + microglia (green) and Olig2+ oligodendrocytes (green) co-stained with EdU (yellow) marker for proliferation in the striatum 24 h after OGD or at equivalent culture time for NC. In all images, cell nuclei are stained with DAPI (blue). Scale bars = 50 μm

An ischemic event in vivo disrupts the brains energy supply and results in neuronal cell death, the extent of which can vary depending on ischemia duration and whether the insult is global or focal. We labeled slices that were co-stained with DAPI and PI with a neuronal marker, anti-NeuN, and used confocal microscopy to collect images from both the cortex and striatum of P10 and P17 slices that received 2 h OGD: representative images are provided in Fig. 7A-B. In all groups, there was overlapping PI and NeuN signal, indicating that a portion of the neuronal population was damaged by OGD, regardless of brain age and region. PI-positive nuclei were also present in areas that contained no NeuN signal, meaning the damage was not selective to mature neurons. In addition to observing an overall increase in EdU+ cells after 24 h OGD, we captured colocalization between Iba1+ microglial cells and EdU as well as between Olig2+ oligodendrocytes and EdU (Fig. 7C). Representative images from the striatum show that EdU+ cells are greater in presence 24 h after 2 h OGD compared to an equivalent culturing time (5DIV) in the NC, and that the proliferating cells are primarily glia.

### Multiple particle tracking (MPT) in the cortex and striatum of P10 and P17 slices following 1 h, 2 h, or 3 h OGD

**Fig. 8:**
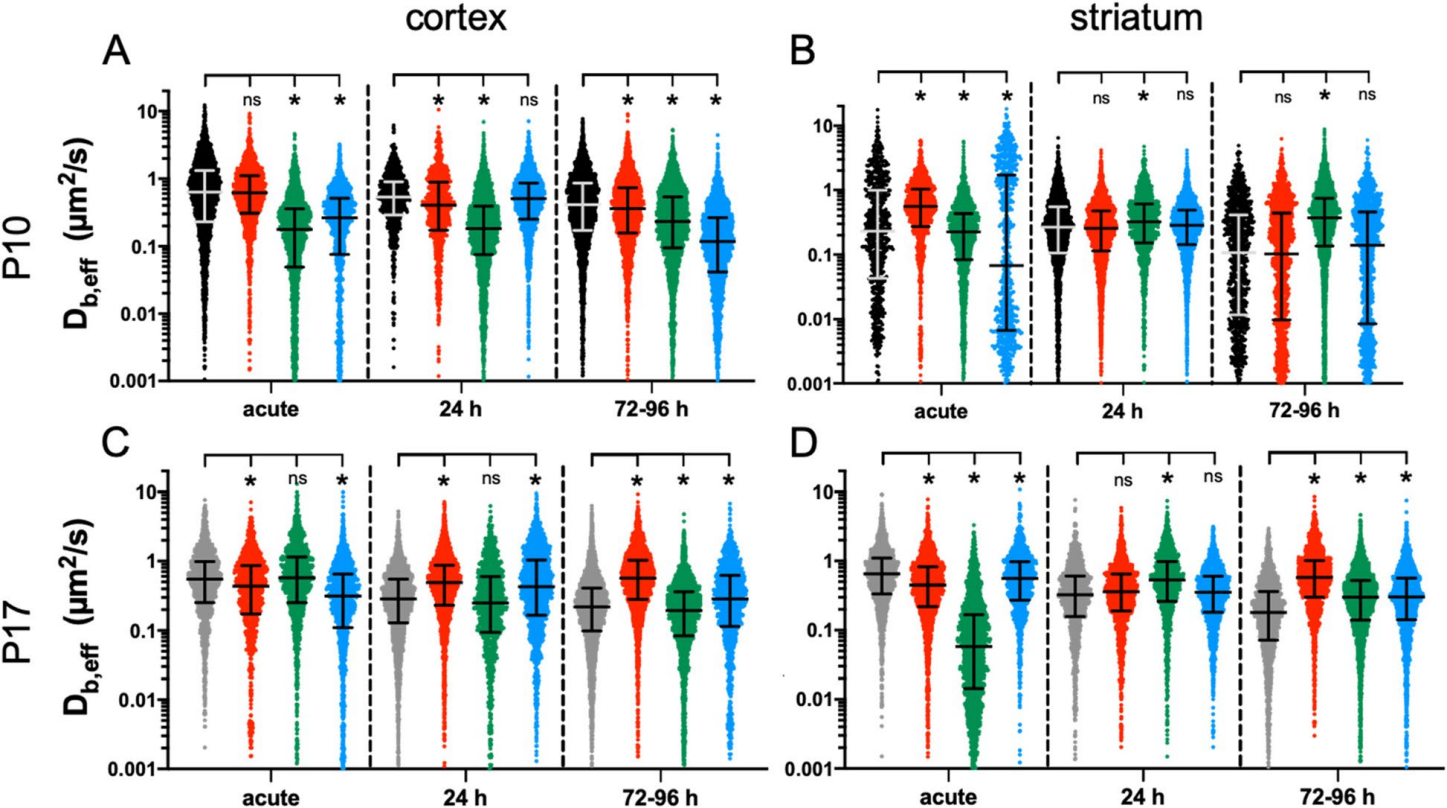
*D*_b,*eff*_ distributions for 40 nm PS-PEG nanoparticles navigating the extracellular space of P10 and P17 brain slices following 1 h, 2 h, or 3 h OGD. *D*_*b,eff*_ distributions were generated from MPT experiments performed at an acute, 24 h-, and 72-96 h post-OGD timepoint. Each row represents a brain age. The top row consists of data generated from the (**A**) cortex and (**B**) striatum of P10 OWH brain slices. The bottom row contains *D*_*b,eff*_ distributions from the (**C**) cortex and (**D**) striatum of P17 OWH brain slices. In all instances, the red, green, and blue markers represent 1 h, 2 h, and 3 h OGD groups, respectively. Error bars represent the median *D*_*b,eff*_ ± IQR

MPT was performed using 40 nm PS-PEG nanoparticles in the cortex and striatum of slices that underwent 1 h, 2 h, or 3 h OGD to evaluate the regional impact of OGD on the extracellular environment. The assessment timepoints were acute (immediately following OGD), 24 h post-OGD, and 72-96 h post-OGD. Distributions of *D*_b,eff_ values at each timepoint, split by brain region and age, are displayed in Fig. 8. Non-OGD-exposed slices were used as the NC.

Almost universally, nanoparticles became more hindered immediately following OGD, regardless of OGD duration, brain region, and age. In all but the striatum of P10 slices, the median *D*_b,eff_ of OGD groups was either not significantly different than or significantly less than the median *D*_b,eff_ in the NC group (Fig. 8B). 1 h OGD in the cortex of P10 slices and 2 h OGD in the cortex of P17 slices were the only other instances where the median *D*_b,eff_ was not significantly reduced following OGD; in these two instances, the median *D*_b,eff_ was not significantly different than the NC (Fig. 8A,C).

The response to OGD became more age-dependent at 24 h and 72-96 h post-OGD. In P10 slices, the impact of OGD on MPT varied regionally. In the cortex of P10 slices, the initial decrease in nanoparticle diffusivity persisted to 24 h and 72-96 h post-OGD. Only the 3 h OGD group at 24 h post-OGD had a median *D*_b,eff_ that wasn’t significantly less than the median *D*_b,eff_ for the NC group (Fig. 8A). By 72-96 h post-OGD, the diffusion of nanoparticles in the cortex of all OGD groups was significantly slower than it was in the NC group, and the impact was OGD-duration-dependent; this trend aligned with an increase in the % of particles that were immobile and a decrease in the % of particles exhibiting normal and superdiffusive behavior (Fig. S3). In the striatum of P10 slices, the response was not as profound at the later timepoints. The only treatment that significantly impacted extracellular nanoparticle diffusion was 2 h OGD, which lead to a significantly greater median *D*_b,eff_ than the NC group at both the 24 h and 72-96 h post-OGD timepoints (Fig. 8B). Lastly, in P10 slices in general, particle tracking in the striatum resulted in a wider distribution of *D*_b,eff_ values than it did in the cortex, especially at the acute and 72-96 h post-OGD timepoints.

In P17 slices, OGD typically led to elevated nanoparticle diffusivities at the later timepoints (24 h and 72-96 h post-OGD). Other than the 2 h OGD group in the cortex, all OGD groups had a significantly greater median *D*_b,eff_ than the NC at the 72-96 h post-OGD timepoint (Fig. 8C-D). In general, P17 slices were more likely than P10 slices to see an immediate decrease in nanoparticle diffusivity following OGD and a subsequent increase in median *D*_b,eff_ values above NC levels at 72-96 h post-OGD, regardless of brain region. The decrease in diffusivity at the acute timepoint coincided with a larger % of particles being immobile and smaller % of particles exhibiting superdiffusive behavior in the striatum of OGD groups (Fig. S4).

## Discussion

OWH brain slices provide direct access to multiple brain regions in a single sample, providing a platform for studying different brain regions in parallel. However, OWH slice models are only useful if cells survive the mechanical trauma and acute hypoxia-ischemia induced by brain slice preparation. Using alamarBlue and LDH assays, we demonstrate an ability to retain viable cells that are metabolically active through 14DIV in OWH slices taken from both P10 and P17 rats. Both age groups saw a decrease in metabolic activity initially, but the metabolism of P10 slices rebounded and increased after 4DIV. An increase in metabolic activity can be on indicator of cell proliferation [51, 52], and EdU imaging and quantification in P10 slices showed there were proliferating cells following explantation. The same increase in metabolic activity was not observed in P17 slices, but metabolism was stable from 4DIV through 14DIV. The combination of a plateau of cumulative LDH release and stable metabolic activity indicates that P17 slices were able to transition into a stable culture state, but P10 slices better handled the transition to ex vivo culturing.

Although not previously shown for OWH slices, this outcome was not unexpected, as brain slice cultures traditionally come from younger donors that contain cell populations more likely to survive explantation [22, 31, 32, 53–58]. While the reasons for better outcomes in neonatal slice preparations are not entirely known, it is thought that the neuronal population in neonates is able to recover faster from hypoxic-ischemic damage [23, 30, 59]. Because of this, it is most common to use donors that are less than 12 days old (< P12). Our qualitative findings show that many PI-positive cells are colocalized with the mature neuron marker, NeuN. Mature neurons are more sensitive to the shearing process and typically require specialized media or culturing conditions to stay viable in culture [29, 60–63], which could impact P17 neuronal health more so than P10 neuronal health if more mature neurons are present at P17.

Whole-slice viability and metabolic activity were also monitored for ten days post-OGD. An extended lack of oxygen and glucose depletes tissue energy reserves and can activate cellular processes that lead to either necrotic or apoptotic cell death. Decreases in the overall number of cells is likely the cause of the reduced metabolic activity seen in our data at longer time points after OGD exposure, given that regardless of OGD duration and donor age, % LDH release at the acute phase of injury was significantly greater than it was in NC slices. In the acute period after an OGD insult, the percentage of EdU+ cells was significantly greater. This suggests OGD causes a response within cells to proliferate more rapidly, which may contribute to the acute metabolic increase after OGD. Microglia [64–66], astrocytes [66, 67], and oligodendrocytes [66, 67] have all been shown to proliferate following hypoxia-ischemia [51, 52]. We observed colocalization between both microglia and oligodendrocytes and EdU, suggesting proliferation of these cells in response to OGD occurs in ex vivo brain slices.

To gain insight into how specific brain regions handled the transition ex vivo, PI staining and imaging revealed that in healthy P10 slices, both the cortex and striatum responded similarly to culturing time. The % of cells damaged in each region was elevated at acute and 1DIV, but by 4DIV*,* the fraction of cells injured was negligible. The damage present at the initial timepoints was expected; it has been well documented that the mechanical trauma induced during slicing damages cells [26, 28, 30]. The PI data suggests that both cortical and striatal tissue taken from the same P10 slice can recover within 4 days and further supports the conclusions drawn from the whole-slice viability and metabolic activity studies. In response to OGD, we also observed variability in different brain regions in both P10 and P17 slices. PI imaging of OGD slices showed that a higher percentage of dead cells were present in the cortex compared to the striatum. This finding aligns with literature that shows brain developmental stage and severity of insult influence the selective regional vulnerability of brain tissue to hypoxia-ischemia [68–70].

To better understand the cellular effect of OGD, we imaged HIF1α, which is involved in cellular response to hypoxia and in regulating proliferation [71]. We observed a significant increase in HIF1α intensity in P10 slices, specifically in the cortex, but not the striatum, 24 h after a 2 h OGD insult. In addition, both the cortex and striatum responded with an increase in percentage of proliferating cells 24 h after OGD, but the response was different temporally. The cortex saw an increase in proliferating cells immediately, but it was not sustained 72 h after OGD. Alternatively, the striatum had a smaller initial increase in proliferating cells, but the response was sustained at 72 h post-OGD. We hypothesize that P10 slices are capable of recovering from injury and that the lack of a sustained effect past 72 h after OGD indicates movement toward normal conditions.

While our findings demonstrate that regional susceptibilities observed in in vivo models of hypoxia-ischemia are retained in ex vivo models of hypoxia-ischemia that use OWH brain slices, the response is brain age-dependent. In healthy P17 slices, despite both LDH release and metabolic activity stabilizing by the end of the 2-week window, PI staining showed a notable fraction of cells still damaged at 14DIV in both the cortex and striatum, and the response was highly variable within each region. PI data generated in P17 OWH slices revealed that the % of cells damaged 72 h post-OGD was significantly lower in OGD groups than it was in the NC, in both brain regions and regardless of OGD duration. We hypothesized microglia would proliferate to aid in clearing the cellular debri [72–74] since they play a significant role in the clearance of dead cells in ex vivo brain slices [27, 75]. However, EdU staining of P17 slices revealed few proliferating cells by 10DIV in the cortex and striatum. The cause of this lack of recovery is difficult to identify, but when considered along with viability and metabolic activity, it suggests that P17 slices have fewer live cells and are not proliferating at a considerable rate. It is also possible that a lower number of dead cells results from the washing steps following PI staining, which would clear the dead cells from the culturing chamber. Future studies could be run to confirm mechanisms of cell clearance; for example, studies investigating microglial contributions to cell clearance could characterize microglia activation state, motility, and dead cell clearance in response to OGD [73–75].

We leveraged MPT, a technique that has been used in combination with brain slices of multiple different species [49, 76, 77], to provide a measure of local extracellular microenvironment changes in normal culturing conditions and in response to OGD. Most existing applications of MPT in brain tissue have focused on altering nanoparticle physicochemical properties to optimize drug delivery properties [49, 78]. In this instance, however, nanoparticle properties were constant and designed to minimize cell uptake in the experimental MPT time frame [49, 50], such that changes in nanoparticle diffusion could unveil changes in the brain environment. One trend that was observed almost universally in both age groups and brain regions was that nanoparticles were most significantly hindered at the initial timepoints following slice preparation or OGD; the extracellular environment became altered in ways that made the ECS more difficult to navigate. The lone exception to this rule was the striatum of P10 slices, where the diffusivity of particle populations was at its greatest immediately following slice preparation, and 1 h OGD lead to a significant increase in diffusivity at the acute timepoint.

There are many potential explanations for the overarching trend of dampened extracellular diffusivity immediately following both slice preparation and OGD. Generally, diffusion can be hindered by steric interactions with cells and ECM structures [5, 79], hydrodynamic interactions brought about by the narrow channels of brain ECS [80, 81], electrostatic interactions with the highly anionic components of brain ECM [82, 83], and hydrophobic interactions between nanoparticle surfaces and proteins present in the ECS. When considering what might contribute to the measurable changes in effective diffusion that we detect, one likely cause is cellular swelling in response to hypoxia, which occurs in both scenarios. Hypoxia, anoxia, and partial or global ischemia are usually accompanied by large ionic shifts in the ECS [66, 84, 85]. OGD rapidly depletes energy stores while compromising the ability to generate ATP via oxidative phosphorylation, disrupts intracellular ion regulation, and drives cellular swelling. Swelling of neurons and glia results in a decrease in the average volume fraction of brain ECS, making it more difficult to navigate the ECS. This phenomenon has been documented previously. ECS volume fraction, 𝛼, has been shown to decrease from its resting value of ~ 0.20 to values as low as 0.05 in in vivo models of severe anoxia or ischemia [17]. The ECS volume fraction reduction has also been seen ex vivo; Pérez-Pinzón et al. found that in adult slices undergoing anoxia, 𝛼 in the cortex decreased from 0.18 to 0.09 [86]. A reduction in extracellular volume fraction from cellular swelling is likely a cause of the decreased nanoparticle diffusivities observed in the P10 and P17 cortex and P17 striatum at all initial timepoints. In almost all instances in our data, the initial reduction in nanoparticle diffusivity gave way to faster moving particle populations. If cellular swelling was the major contributor to hindered diffusion at the initial timepoints, then as the initial damage induced by slicing subsides, cellular edema should also revert and in turn 𝛼 will return to a more normal value of ~ 20% [87–89]. The elevated cellular damage observed in P17 slices compared to P10 slices also provides an explanation as to why the trends in extracellular nanoparticle diffusion were more pronounced in P17 tissue.

One major difference between slice preparation and OGD is the mechanical trauma associated with shearing brain tissue to generate slices. This mechanical trauma could also be altering the extracellular microenvironment in ways that affect extracellular diffusion, on top of the impact of hypoxia-induced cellular swelling. In models of traumatic brain injury, effective diffusivity in brain ECS has been shown to decrease significantly following injury, even if a reduction in 𝛼 is not present [90]. This increase in diffusional hindrance is thought to be caused by increased expression and accumulation of ECM components, such as hyaluronic acid and CSPGs, in the ECS. There is potential that in our NC slices both ECM protein accumulation and cellular swelling are combining to reduce nanoparticle diffusivity at the acute and 1DIV culturing timepoints. Altered ECM composition and structure caused by glial activation also likely contribute to the elevated diffusivities observed at 24 h and 72-96 h post-OGD. Glial migration and proliferation in response to hypoxic-ischemic events [91, 92], which we also show in our data in response to OGD, has been associated with both the down-regulation of ECM protein expression [93, 94] and increased expression of ECM degrading matrix metalloproteinases [95, 96]. Changes in brain ECM structure and composition are known to impact the extracellular diffusion of both small molecules [83, 97] and nano-sized probes [19, 98]. Future work focused on quantifying ECM protein expression through 14DIV and following OGD would provide clarity.

Alternatively, the increase in nanoparticle diffusivity at later times after OGD could be a result of cell death and subsequent clearance. Clearance of these dead cells would result in decreased cell densities, provide more void spaces for nanoparticles to diffuse, and lead to faster moving particle populations. The increase in diffusivity at the later stages of OGD injury aligns with previous diffusion-weighted magnetic resonance imaging (DW-MRI) studies that found increases in both the apparent diffusion coefficient of water (ADC_w_) and 𝛼 at later phases of reperfusion [66]. OGD insult is known to induce significant neuronal damage that can result in cell death, which we were able to show through elevated LDH release, elevated PI signal, reduced metabolic activity, and PI-NeuN co-staining. These localized structural disintegrations can give rise to elevated diffusivity [99, 100].

Given that we observe significant fluctuations in median *D*_*b,eff*_ values at the later stages of the 2-week culture window (4DIV–14DIV) in both the striatum and cortex of NC P10 brain slices, we also must consider the contribution of biological variability. Although all studies were done in females in this work, future work should aim to incorporate additional regions, sex, and ages to assess variability in the extracellular response to donor age and subsequent OGD exposure. Lastly, one factor that should be considered is the effect of slice thinning over culture time. Organotypic hippocampal slice thickness will decrease throughout the first week in vitro, regardless of starting thickness [101] and different donor ages and different brain regions may thin at different rates. The exact impact thinning has on the organization of the cells in culture is not fully understood, but if it were to significantly affect either cell density or the local extracellular environment, it would likely impact the behavior of extracellularly diffusing nanoparticles.

## Conclusions

We were able to maintain viable, metabolically active OWH brain slices taken from both P10 and P17 rats through two weeks in vitro*,* but found that P10 slices respond better to explantation. While whole-slice LDH release and metabolic activity stabilized in P17 slices by 14DIV, damaged cells were still present in both the cortex and striatum. Cultures established from older slices could provide ex vivo models that better represent a more mature and developed brain, specifically regarding neuronal and glial organizational patterns and phenotypes that better represent mature neurons [30]. The ability to keep multiple brain regions viable in a single sample allowed us to evaluate if striatal and cortical tissue responded differently to either the transition ex vivo or OGD. EdU staining demonstrated that P10 slices have actively proliferating cells, and more so in the striatum than the cortex. The percentage of proliferating cells is increased after OGD, but the duration of response is dictated by brain region. HIF1α staining also had a higher intensity after OGD than NC, which confirms OGD induces hypoxia in ex vivo brain slices. MPT revealed that the extracellular environment was most difficult for nanoparticles to navigate either immediately after or the day following explantation, when cell death was increasing at the highest rate. As the initial damage induced by slicing subsided, brain tissue became easier to diffuse in, in general. OGD immediately elevated whole-slice % LDH release in both P10 and P17 slices, but the % of cells damaged was age- and region-dependent. Similarly, the impact OGD had on the extracellular environment of brain slices was both age- and region-dependent and is likely a result of the selective regional vulnerability of brain tissue to ischemia. OGD led to a decrease in nanoparticle diffusivity initially, but the extracellular environment became easier to navigate both 24 h- and 72-96 h post-OGD. Additional studies tailored to more comprehensively characterize brain ECM composition and brain ECS properties would provide further clarity towards specific mechanisms that alter extracellular diffusion in response to OGD. Collectively, this work establishes a viable OWH brain slice platform for studying the impact of biological variables, such as age and anatomical region, and injury on the brain cellular and extracellular environment.

## Methods

### Animal work and ethics statement

All animal work was performed in accordance with the recommendations in the Guide for the Care and Use of Laboratory Animals of the National Institutes of Health (NIH). Animals were handled according to approved Institutional Animal Care and Use Committee (IACUC) protocol (#4383–02) of the University of Washington, Seattle, WA, USA. The University of Washington has an approved Animal Welfare Assurance (#A3464–01) on file with the NIH Office of Laboratory Animal Welfare (OLAW), is registered with the United States Department of Agriculture (USDA, certificate #91-R-0001), and is accredited by AAALAC International. Every effort was made to minimize suffering. All work was performed using Sprague-Dawley (SD) rats (virus antibody-free CD®, *Rattus norvegicus*, IGS, Charles River Laboratories, Raleigh, NC, USA) that arrived at P5 with a nursing dam. Before removal from the dam at P10 or P17, each dam and her pups were housed under standard conditions with an automatic 12 h light/dark cycle, temperature range of 20–26 °C, and access to standard chow and sterile tap water ad libitum*.* The pups were checked for health daily. To eliminate the influence of sex-based differences, all animals used were female.

### Preparation of organotypic whole hemisphere (OWH) brain slice cultures

Healthy female SD rats were injected with an overdose of 100 μL pentobarbital (Commercial Beuthanasia D, 390 mg/mL pentobarbital, administered > 120–150 mg/kg) intraperitoneally. Once the animal was unresponsive to a toe pinch with tweezers, it was decapitated with surgical scissors. The brain was removed rapidly under aseptic conditions and submerged in ice cold dissection media consisting of 100% HBSS (Hank’s Balanced Salt Solution, no Mg^2+^, no Ca^2+^, Thermo Fisher Scientific, Waltham, MA, USA), 1% Penicillin-Streptomycin (Thermo Fisher Scientific), and 0.64% w/v glucose (MilliporeSigma, Burlington, MA, USA). Whole brains were split into hemispheres with a sterile razor blade and sliced coronally into 300 μm-thick sections with a Mcllwain tissue chopper (Ted Pella, Inc., Redding, CA, USA). Individual slices were separated in ice cold dissection media using sterile fine tip paint brushes and transferred onto 30-mm 0.4-μm-pore-sized cell culture inserts (hydrophilic PTFE, MilliporeSigma) before being placed in a non-treated 6-well plate (USA Scientific Inc., Ocala, FL, USA) containing 1 mL pre-warmed (37 °C) slice culture media (SCM; 50% MEM [minimum essential medium, no glutamine, no phenol red, Thermo Fisher Scientific], 21.75% HBSS [with Mg^2+^, with Ca^2+^], 25% horse serum [heat inactivated, New Zealand origin, Thermo Fisher Scientific], 1.25% HEPES [Thermo Fisher Scientific], 0.575% w/v glucose, 1% GlutaMAX Supplement, and 1% Penicillin-Streptomycin). Slices were cultured in a sterile CO_2_ incubator (Thermo Fisher Scientific) at 37 °C with constant humidity, 95% air and 5% CO_2_.

All slices were collected at a depth that provided access to both the cerebral cortex and striatum. SCM exchanges were performed 3–4 h after slice preparation, then subsequently on 1, 4, 7, 11, and 14-days in vitro (DIV). All media added to slices was pre-warmed to 37 °C. For alamarBlue (aBlue) and lactate dehydrogenase (LDH) assays, 2 slices were plated per insert. In all other instances, 1 brain slice was plated per insert.

### AlamarBlue (aBlue) assay for OWH brain slice relative metabolic activity

Two brain slices were plated per insert. aBlue working reagent was prepared by diluting 10x stock solution (alamarBlueTM Cell Viability Reagent, Thermo Fisher Scientific) 1:10 in pre-warmed (37 °C) SCM. aBlue working reagent was always made fresh. To initiate an experiment, brain slices were removed from the incubator and the existing SCM was discarded and replaced with 1 mL aBlue working reagent. Brain slices were returned to the incubator and allowed to incubate for 24 h. Slices were then removed from the incubator and the aBlue working reagent was collected. Slices were washed twice for 3 min each with 1 mL room temperature 1xPBS then replenished with 1 mL pre-warmed (37 °C) SCM and returned to the incubator for subsequent time points. The aBlue working reagent was run immediately by loading 100 μL in a non-treated 96-well plate and measuring the 560/590 nm excitation/emission fluorescence on a Synergy H1 multimode microplate reader (BioTek, Winooski, VT, USA). Samples were run in triplicate and the average value was reported. For P10 and P17 normal control (NC) studies comparing different days in vitro (DIV), readings were normalized to the mean metabolic activity at the acute time point (set at 100%). For OGD studies, readings were normalized to the mean metabolic activity of the corresponding normal control group and the % difference from NC was reported. A negative metabolic activity control was carried out by treating 14 DIV slices with 1% Triton X-100 (TX-100, MilliporeSigma) in SCM for 2 h prior to starting the 24 h incubation with aBlue working reagent.

### Lactate dehydrogenase (LDH) assay for OWH brain slice viability

Two brain slices were plated per insert. For P10 and P17 normal NC studies, SCM supernatant was collected at an acute (3–4 h) timepoint, then subsequently on 1 DIV, 4 DIV, 7 DIV, 11 DIV, and 14 DIV. To generate a positive cell death control, slices at 14 DIV were treated for 2 h with 1% TX-100 in SCM. The supernatant was collected following TX-100 treatment. All supernatant samples were immediately stored at − 80 °C. Supernatant samples were removed and thawed at room temperature to conduct LDH assays (601,170, Cayman Chemical, Ann Arbor, MI, USA). Following the manufacturer’s instructions, 100 μL of LDH reaction buffer was added to 100 μL of sample supernatant in triplicate to 96-well plates on ice, and the plates were transferred to a stir plate in a 37 °C incubator. After 30 min, absorbance at 490 nm was measured on a Synergy H1 multimode microplate reader to detect the production of colorimetric formazan. All LDH readings were normalized to an acute positive cell death control. Percent LDH release was calculated using the following equation:

$$\%\mathrm{LDH}\ \mathrm{release}=\frac{cumulative\ LDH\ absorbance\ of\ sample}{LDH\ absorbance\ of\ TX\ 100\ acute\ sample}$$

### Propidium iodide (PI) staining of OWH brain slices for % cell damage

At specified timepoints, slices were stained with 1 mL of 5 μg/mL PI (Thermo Fisher Scientific) in SCM for 45 min at standard culture conditions. The staining solution was placed underneath the insert. Slices were washed twice for 3 min each with sterile 1xPBS at room temperature, followed by a 1 h wash with 37 °C SCM at culturing conditions, and then formalin fixed for 1 h with 10% formalin phosphate buffer (Thermo Fisher Scientific). Following two final washes with room temperature 1xPBS, slices were stored covered at 4 °C until ready for use. Within 2 weeks, slices were stained for 30 min with 0.1 μg/mL DAPI (Thermo Fisher Scientific) in 1xPBS at room temperature. Slices were washed twice for 3 min each with 1xPBS prior to imaging. Two-channel 40x confocal images (oil immersion, 1.30 numerical aperture, Nikon Corporation, Minato City, Tokyo, Japan) were obtained for PI and DAPI. For every slice, five images were acquired from both the cortex and striatum. Image acquisition settings were consistent for all images and conditions. For each image, DAPI+ cell nuclei (total cells) and PI+ cell nuclei (dead cells) that were also DAPI+ were counted manually in ImageJ (NIH) after applying an Otsu threshold and fluorescent cutoff to aid in visualization. The fluorescent cutoff was kept consistent across all images. The PI+/DAPI+ cell ratio was expressed as the percentage of dead cells in an individual image.

### Immunofluorescence staining in fixed OWH brain slices

Brain slices were prepared, stained with PI, and formalin fixed in the same manner they were for PI staining and imaging studies. Following fixation, slices were incubated with primary antibodies for NeuN (neurons), H1F1α, Iba1 (microglia), or Olig2 (Oligodendrocytes). On fixed slices, AlexaFluor® 647 Anti-NeuN (rabbit anti-NeuN, Abcam Cat # ab190565, Cambridge, UK) at a 1:250 dilution in 1x PBS containing 1% Triton X-100 and 3% donkey serum (MilliporeSigma, Cat # S30-100 mL) was applied overnight at 4 °C. Following a 1x PBS wash step, cellular nuclei were stained with a 0.1 μg/mL solution of DAPI in 1x PBS for 15 min. Following a final wash, slices were stored at 4 °C until they were imaged. For H1F1α, after 1 h in 0.5% Triton X-100, slices were washed, and primary antibody for H1F1α was added at 1:250 in TBST with 5% goat serum overnight at 4 °C. (ThermoFisher Scientific, Cat # PA1–16601). After washing with additional TBST, goat anti-rabbit AlexaFluor® 594 (ThermoFisher Scientific, Cat # A-11012) was added at 1:500 for 2 h in identical conditions to primary. Slices were washed with PBS and stored at 4 °C until imaging. For Iba1, slices were incubated overnight at 4 °C in anti-Iba1 (rabbit anti-Iba, Wako Cat # 019–19,741) at 1:250 dilution in 1x PBS containing 1% Triton X-100 and 3% donkey serum and washed as described for NeuN. For anti-Olig2, slices were incubated overnight at 4 °C in anti-olig2 (rabbit anti-olig2, Abcam Cat # ab109186) at 1:250 dilution in 1x PBS containing 1% Triton X-100 and 3% donkey serum and washed as described for NeuN. For both Iba1 and Olig2 stained slices, slices were stained with an AlexaFluor® 488 goat anti-rabbit secondary antibody (ThermoFisher Scientific Cat # A11034) at 1:500 dilution for 2 h in the same buffer conditions as the primary antibody. Following a 1x PBS wash step, cellular nuclei were stained with a 0.1 μg/mL solution of DAPI in 1x PBS for 15 min. Following a final wash, slices were stored at 4 °C until they were imaged.

### EdU cell proliferation assay and analysis

For proliferation analysis via EdU staining, a kit from Click Chemistry Tools was used according to recommended conditions (ClickChemistryTools, Cat # 1329). Briefly, EdU was added to live brain slices via culture media at 20 μm for 16 hours. Following fixation, an azide-conjugated dye was reacted with EdU labeled cells in the presence of copper catalyst for 1 h. Following a 1x PBS wash step, cellular nuclei were stained with a 0.1 μg/mL solution of DAPI in 1x PBS for 30 min prior to imaging. Three images were acquired at 40x magnification using 405 and 647 laser lines in the cortex and striatum. Each experimental group contained 3 brain slices and image acquisition settings were kept constant throughout. An ImageJ macro was used to set consistent LUT values prior to manual counting of total cells positive for DAPI and total cells positive for EdU in acquired images. The ratio of these values (EdU/DAPI) represents the percentage of proliferating cells. Researchers were blinded during imaging and manual cell counting. Statistical analysis was performed in GraphPad Prism.

### HIF1α imaging and analysis

Slices were stained for HIF1α as described above. Three images were acquired at 40x magnification using 405 and 647 laser lines in the cortex and striatum. Each experimental group contained 3 brain slices and image acquisition settings were kept constant throughout. To further minimize variability, all slices were imaged in the same session. A macro created in ImageJ was used to measure the mean pixel intensity of HIF1α and DAPI in each image. The mean pixel intensity of HIF1α was normalized using the mean pixel intensity of DAPI. Researchers were blinded during imaging and recording mean pixel value measurements in ImageJ. Statistical analysis was performed in GraphPad Prism.

### Oxygen-glucose deprivation (OGD) of OWH brain slices

OGD media consisted of 120 mM sodium chloride (NaCl, MilliporeSigma), 5 mM potassium chloride (KCl, MilliporeSigma), 2 mM calcium chloride (CaCl_2_, MilliporeSigma), 1.25 mM monosodium phosphate anhydrous (NaH_2_PO_4_, MilliporeSigma), 2 mM magnesium sulfate (MgSO_4_, MilliporeSigma), 25 mM sodium bicarbonate (NaHCO_3_, MilliporeSigma), and 20 mM HEPES in DI water. OGD media was sterile filtered (0.2 μm), titrated to pH 7.4 with 1 M hydrochloric acid (Thermo Fisher Scientific) or 1 M sodium hydroxide (Thermo Fisher Scientific), bubbled with nitrogen gas (Praxair, Danbury, CT, USA) for at least 10 min, and pre-warmed to 37 °C prior to use. To initiate OGD, the SCM supernatant was removed, and each well was rinsed once with 1 mL OGD media then replenished with 1 mL fresh OGD media. The membrane inserts were then placed back in the well. The 6-well plates with OGD samples were placed in a Hypoxia Incubator Chamber (STEMCELL Technologies, Vancouver, Canada) and placed in a 37 °C incubator. The chamber was flushed with nitrogen gas for at least 10 min then clamped shut to prevent O_2_ from entering. Slices were incubated in the chamber for the remainder of their treatment duration (1 h, 2 h, or 3 h). Following OGD, wells were rinsed once with SCM then replenished with 1 mL fresh SCM. The 6-well plates were then returned to standard culture conditions. Evaluation timepoints occurred at either an acute timepoint (immediately after the OGD session), 24 h post-OGD, or 72–96 h post-OGD. Supernatants were collected and assessed using LDH assay. Slices were stained for PI, EdU, Iba1, NeuN, Olig2, and HIF1α.

### Nanoparticle preparation and characterization

Fluorescent carboxylate (COOH)-modified polystyrene latex (PS) nanoparticles (PS-COOH) (Thermo Fisher Scientific) were covalently modified with methoxy (MeO)-poly(ethylene glycol) (PEG)-amine (NH_2_) (5 kDa MW, Creative PEGWorks, Durham, NC, USA) by carboxyl amine reaction [102]. Briefly, 50 μL of PS-COOH particle suspension was diluted 6-fold in deionized (DI) water. MeO-PEG-NH_2_ was added to stoichiometrically complement the number of -COOH present on the particle surface. *N*-Hydroxysulfosuccinimide (NHS, MilliporeSigma) was added at a 10-fold molar excess of PEG, the solution was vortexed briefly, then 1200 μL of 200 mM borate buffer, pH 8.2, was added. 1-Ethyl-3-(3-dimethylaminopropyl) carbodiimide (EDC, Thermo Fisher Scientific) was added to stoichiometrically complement the NHS. Particle suspensions were placed on a rotary incubator for 24 h at 25 °C then washed via centrifugation (Amicon Ultra 0.5 mL 100 k MWCO; MilliporeSigma) at conditions specified previously [102]. Particles were resuspended in DI water to the initial particle volume and stored at 4 °C until use. The hydrodynamic diameter and polydispersity index (PDI) of the resulting PEG-conjugated fluorescent nanoparticles were measured via dynamic light scattering (DLS) and the ζ-potential by laser Doppler anemometry. Both DLS and laser Doppler anemometry were performed using the Zetasizer Nano ZS (Malvern Panalytical, Malvern, UK). Particles were diluted to ~ 0.002% solids in filtered (0.45-um, Whatman, Maidstone, UK) 10 mM NaCl, pH 7.0, prior to measurement.

### Multiple particle tracking (MPT) of 40 nm PS-PEG nanoparticles in OWH brain slice cultures

For MPT experiments, one brain slice was plated per insert. At specified timepoints, SCM was exchanged for 1 mL prewarmed (37 °C) SCM containing 40 nm PS-PEG nanoparticles (100 μg/mL) and Hoechst (5 drops/mL, NucBlue Live ReadyProbes Reagent, Hoechst 33342, Thermo Fisher Scientific). 900 μL of nanoparticle-Hoechst-SCM solution was added below the insert and 100 μL was added dropwise to the top of the slice. Slices were incubated for 1 h at standard culture conditions then washed twice with 1 mL warm (37 °C) SCM for 3 min each. Following the second wash, slices were transferred to an imaging dish and kept in a temperature-controlled incubation chamber maintained at 37 °C, 5% CO_2_, and 80% humidity during the imaging session. Video acquisition was completed within 2 h. One video was collected and quantified from the cortex and striatum of each slice. Videos were collected at 33.3 frames-per-second and 100x magnification (oil immersion, 1.45 numerical aperture, Nikon Corporation) for 651 frames via fluorescent microscopy using a cMOS camera (Hamamatsu Photonics, Hamamatsu City, Japan) mounted on a confocal microscope.

### Multiple particle tracking (MPT) analysis

Nanoparticle trajectories, trajectory MSDs, and D_eff_ (or D_b,eff_) values were extracted from microscopy videos via a lab-developed Python package diff_classifier for parallelized and reproducible MPT workflows (https://github.com/Nance-Lab/diff_classifier) [47]. The TrackMate plugin generates segmented trajectories for each video that provide particle positions in the x- and y-dimension at each frame of the video. From this, geometrically-averaged precision-weighted trajectory mean squared displacements (<MSD>) were calculated for each trajectory and timestep using the equation:

$$<{r}^2>=\frac{1}{N-1}{\sum}_{i=0}^{N-n-1}{\left|{x}_{i+N}-{x}_i\right|}^2$$

where *r*^2^ indicates the MSD determined at each step, *n*, for a total number of steps, *N*, with 3D position coordinates *x* (*x*, *y*, *t*). Effective diffusion coefficients, D_eff_ (or D_b,eff_ when videos were acquired in brain tissue), were calculated using the Einstein-Smoluchowski Equation for two dimensional diffusion:

$${D}_{eff}=\frac{MSD}{4t}$$

where *t* is the time interval between which particle positions are observed. To calculate anomalous diffusion exponent (α), MSD curves were fit to the anomalous diffusion equation:

$$MSD=4{D}_{eff}{\tau}^a-$$

where *τ* is lag time.

### Statistical analysis

All statistical analyses were carried out in GraphPad Prism (GraphPad Software Inc). For all tests run, differences were defined as statistically significant at *p* < 0.05. The D’Agostino-Pearson omnibus K2 test was used to test for normality. If we were unable to reject the null hypothesis that data were sampled from a population that follows a Gaussian distribution, we either ran an ordinary one-way ANOVA or Brown-Forsythe and Welch ANOVA test, depending on if we could assume equal standard deviations. In instances where an ordinary one-way ANOVA was performed and multiple comparisons were made, we used Tukey’s multiple comparisons test. In instances where the Brown-Forsythe and Welch ANOVA test was performed and multiple comparisons were made, we used Dunnett’s T3 multiple comparisons test. If we were able to reject the null hypothesis that the data were taken from a normally distributed population, we used the Kruskal-Wallis test for significance. In these instances, we applied Dunn’s method to correct for multiple comparisons.

## Supplementary Information

**Additional file 1: Table S1.** Physicochemical properties of the 40 nm PS-PEG nanoparticles. Nanoparticle hydrodynamic diameter and PDI as determined by DLS. Laser Doppler anemometry was used to determine nanoparticle ζ-potential. All experiments were performed at 25 °C in 10 mM NaCl, pH 7.0. Values represent the average ± standard deviation of *n* = 3 measurements. **Fig. S1.** Percentage of proliferating cells (EdU+) evaluated at 10 DIV. A The overall cell proliferation data for P10 and P17 slices. B The percentage of proliferating cells by region. Each point is obtained from an image, and images are obtained from 3 total slices (*n* = 3) per group. **Fig. S2.** Fraction of particles exhibiting either immobile, subdiffusive, normal diffusive, or superdiffusive behavior in P10 and P17 NC slices over the first 14 days in vitro. Trajectories were assigned to each diffusion group depending on their fitted anomalous diffusion exponent, α. The top row consists of data collected from the (A) cortex and (B) striatum of P10 slices. The bottom row consists of data collected from the (A) cortex and (B) striatum of P17 slices. **Fig. S3.** Fraction of particles exhibiting either immobile, subdiffusive, normal diffusive, or superdiffusive behavior after 1 h, 2 h, and 3 h OGD in P10 slices. Trajectories were assigned to each diffusion group depending on their fitted anomalous diffusion exponent, α. Each row represents an assessment timepoint. Each column represents a region. The top row consists of data collected from the acute timepoint and is split between (A) cortex and (B) striatum. The middle row consists of data from the 24 h post-OGD timepoint and is split between (C) cortex and (D) striatum. Data generated from the (E) cortex and (F) striatum at 72-96 h post-OGD is provided in the third, final row. **Fig. S4.** Fraction of particles exhibiting either immobile, subdiffusive, normal diffusive, or superdiffusive behavior after 1 h, 2 h, and 3 h OGD in P17 slices. Trajectories were assigned to each diffusion group depending on their fitted anomalous diffusion exponent, α. Each row represents an assessment timepoint. Each column represents a region. The top row consists of data collected from the acute timepoint and is split between (A) cortex and (B) striatum. The middle row consists of data from the 24 h post-OGD timepoint and is split between (C) cortex and (D) striatum. Data generated from the (E) cortex and (F) striatum at 72-96 h post-OGD is provided in the third, final row.

## Data Availability

The datasets supporting the conclusions of this article are available upon request. Please contact the corresponding author for access. All code is available on github, with links included in the methods.
