# Supplementary material for: Organotypic whole hemisphere brain slice models to study the effects of donor age and oxygen-glucose-deprivation on the extracellular properties of cortical and striatal tissue
Source: J Biol Eng. 2022 Jun 13;16:14. doi: 10.1186/s13036-022-00293-w (PMC9195469; doi:10.1186/s13036-022-00293-w)
Supplement: Supplementary file 1 — Additional file 1: Table S1. Physicochemical properties of the 40 nm PS-PEG nanoparticles. Nanoparticle hydrodynamic diameter and PDI as determined by DLS. Laser Doppler anemometry was used to determine nanoparticle ζ-potential. All experiments were performed at 25 °C in 10 mM NaCl, pH 7.0. Values represent the average ± standard deviation of n = 3 measurements. Fig. S1. Percentage of proliferating cells (EdU+) evaluated at 10 DIV. A The overall cell proliferation data for P10 and P17 slices. B The percentage of proliferating cells by region. Each point is obtained from an image, and images are obtained from 3 total slices (n = 3) per group. Fig. S2. Fraction of particles exhibiting either immobile, subdiffusive, normal diffusive, or superdiffusive behavior in P10 and P17 NC slices over the first 14 days in vitro. Trajectories were assigned to each diffusion group depending on their fitted anomalous diffusion exponent, α. The top row consists of data collected from the (A) cortex and (B) striatum of P10 slices. The bottom row consists of data collected from the (A) cortex and (B) striatum of P17 slices. Fig. S3. Fraction of particles exhibiting either immobile, subdiffusive, normal diffusive, or superdiffusive behavior after 1 h, 2 h, and 3 h OGD in P10 slices. Trajectories were assigned to each diffusion group depending on their fitted anomalous diffusion exponent, α. Each row represents an assessment timepoint. Each column represents a region. The top row consists of data collected from the acute timepoint and is split between (A) cortex and (B) striatum. The middle row consists of data from the 24 h post-OGD timepoint and is split between (C) cortex and (D) striatum. Data generated from the (E) cortex and (F) striatum at 72-96 h post-OGD is provided in the third, final row. Fig. S4. Fraction of particles exhibiting either immobile, subdiffusive, normal diffusive, or superdiffusive behavior after 1 h, 2 h, and 3 h OGD in P17 slices. Trajectories were assigned to e [file 13036_2022_293_MOESM1_ESM.docx]

Supplemental Information

Table S1. Physicochemical properties of the 40nm PS-PEG nanoparticles.

Nanoparticle hydrodynamic diameter and PDI as determined by DLS. Laser Doppler anemometry was used to determine nanoparticle ζ-potential. All experiments were performed at 25°C in 10mM NaCl, pH 7.0. Values represent the average ± standard deviation of n=3 measurements.

| **Sample** | **Hydrodynamic Size (nm)** | **PDI** | **𝜁-Potential (mV)** |
| --- | --- | --- | --- |
| 40nm PS-PEG | 50.3 ± 1.5 | 0.06 ± 0.01 | -1.17 ± 0.34 |


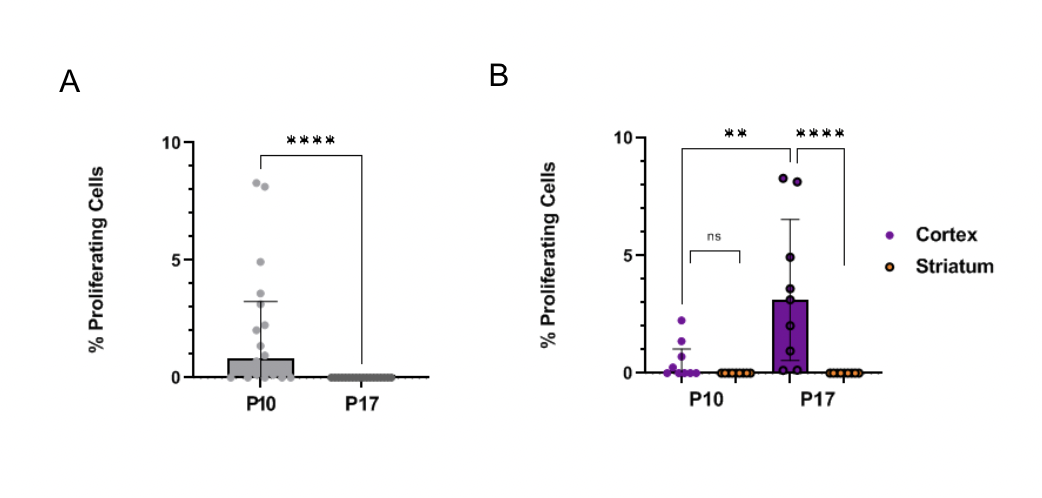


Figure S1. Percentage of proliferating cells (EdU+) evaluated at 10 DIV. (A) The overall cell proliferation data for P10 and P17 slices. (B) The percentage of proliferating cells by region. Each point is obtained from an image, and images are obtained from 3 total slices (n=3) per group.


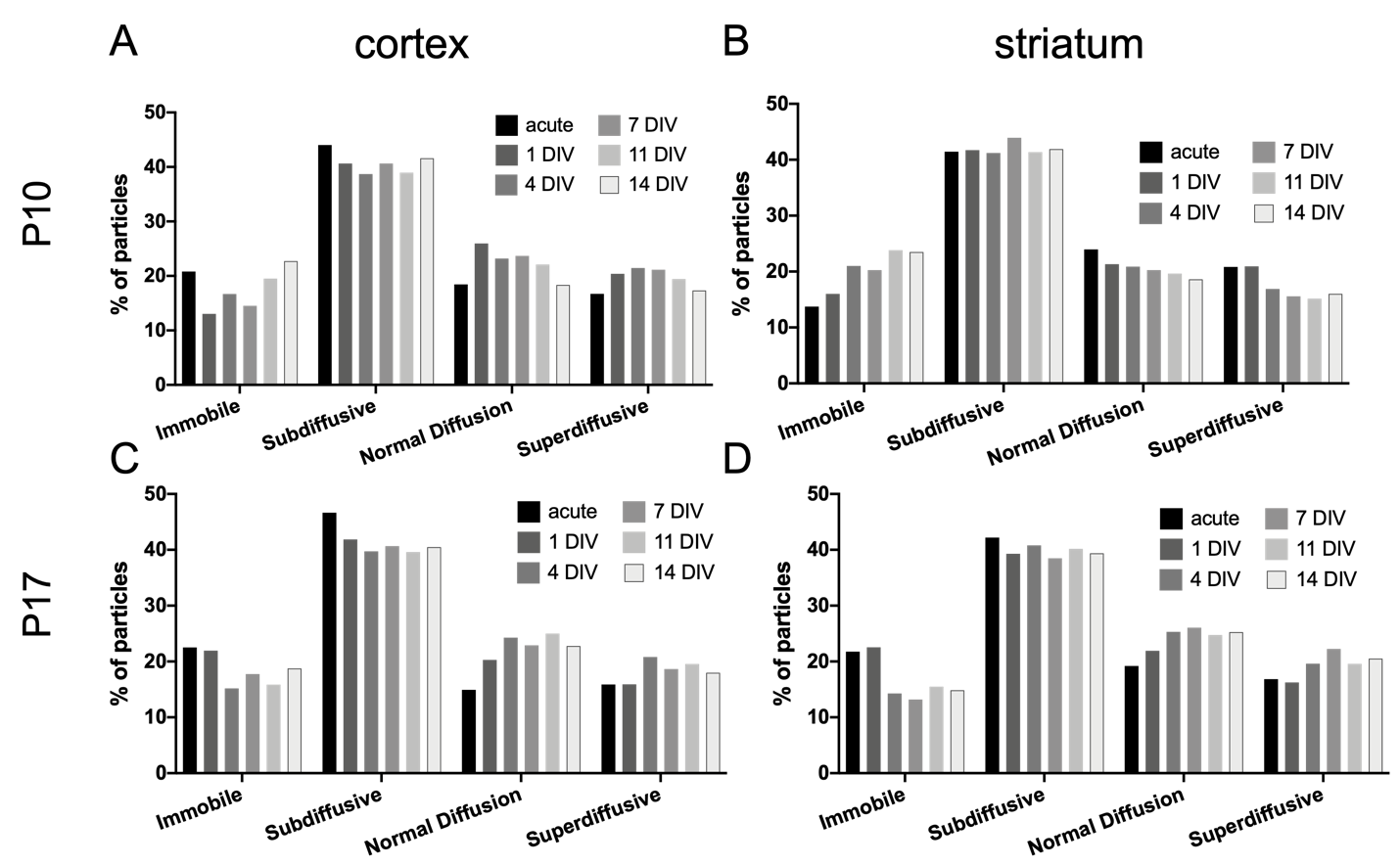


Figure S2. Fraction of particles exhibiting either immobile, subdiffusive, normal diffusive, or superdiffusive behavior in P10 and P17 NC slices over the first 14 days in vitro.

Trajectories were assigned to each diffusion group depending on their fitted anomalous diffusion exponent, α. The top row consists of data collected from the (A) cortex and (B) striatum of P10 slices. The bottom row consists of data collected from the (A) cortex and (B) striatum of P17 slices.


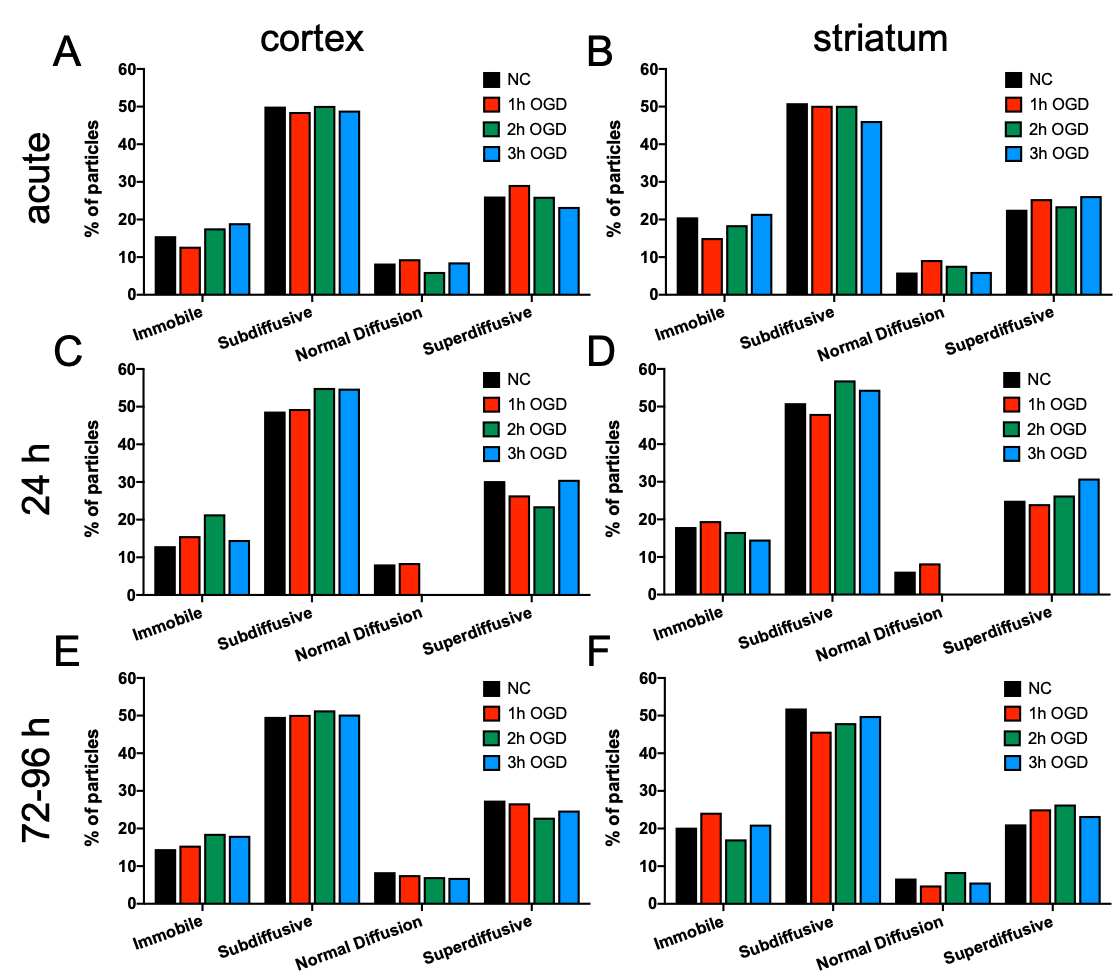


Figure S3. Fraction of particles exhibiting either immobile, subdiffusive, normal diffusive, or superdiffusive behavior after 1h, 2h, and 3h OGD in P10 slices.

Trajectories were assigned to each diffusion group depending on their fitted anomalous diffusion exponent, α. Each row represents an assessment timepoint. Each column represents a region. The top row consists of data collected from the acute timepoint and is split between (A) cortex and (B) striatum. The middle row consists of data from the 24h post-OGD timepoint and is split between (C) cortex and (D) striatum. Data generated from the (E) cortex and (F) striatum at 72-96h post-OGD is provided in the third, final row.


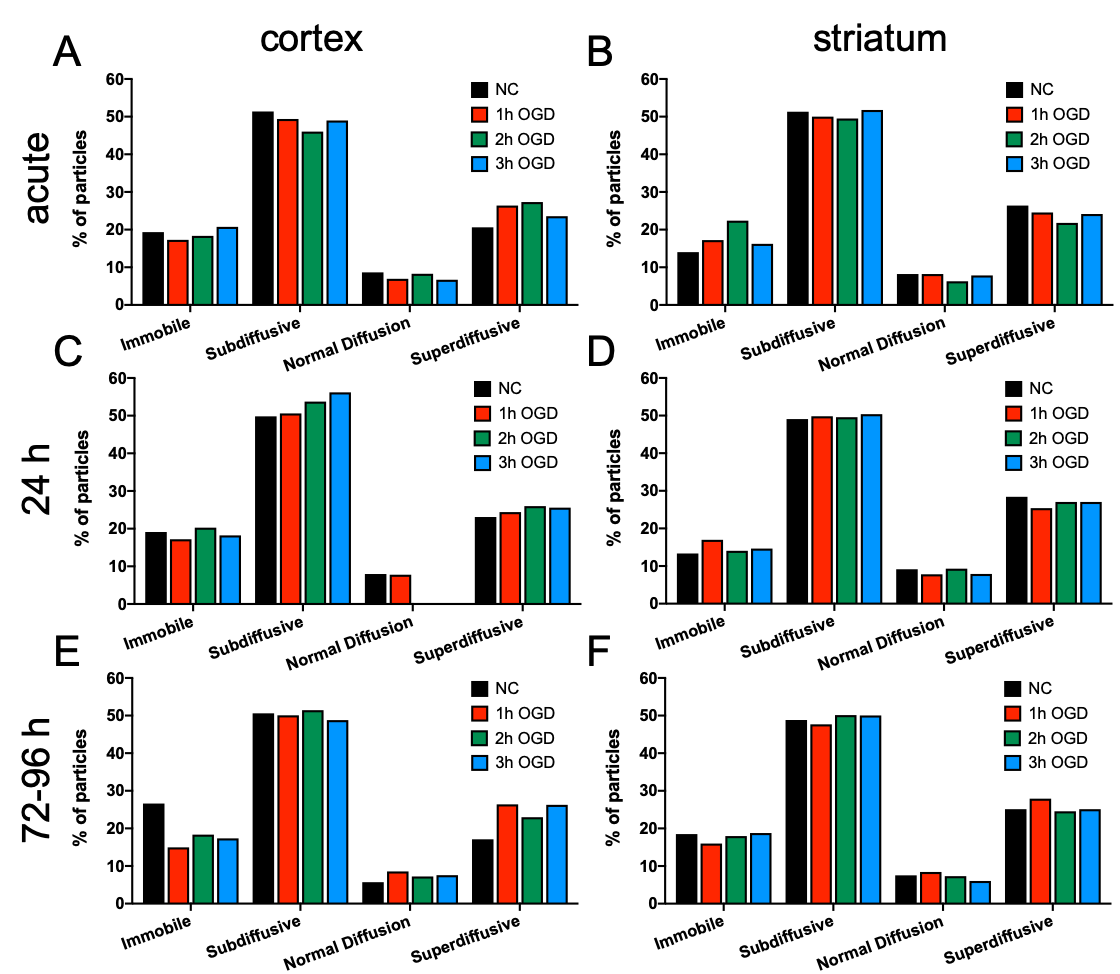


Figure S4. Fraction of particles exhibiting either immobile, subdiffusive, normal diffusive, or superdiffusive behavior after 1h, 2h, and 3h OGD in P17 slices.

Trajectories were assigned to each diffusion group depending on their fitted anomalous diffusion exponent, α. Each row represents an assessment timepoint. Each column represents a region. The top row consists of data collected from the acute timepoint and is split between (A) cortex and (B) striatum. The middle row consists of data from the 24h post-OGD timepoint and is split between (C) cortex and (D) striatum. Data generated from the (E) cortex and (F) striatum at 72-96h post-OGD is provided in the third, final row.
